# Covalent Modification by Click Mechanochemistry: Systematic Installation of Pendant OH Groups in a MOF for Rigidity Control and Luminescence-Based Water Detection

**DOI:** 10.1021/acsami.3c00788

**Published:** 2023-05-19

**Authors:** Damian Jędrzejowski, Michał Ryndak, Jakub J. Zakrzewski, Maciej Hodorowicz, Szymon Chorazy, Dariusz Matoga

**Affiliations:** † Faculty of Chemistry, Jagiellonian University in Kraków, Gronostajowa 2, 30-387 Kraków, Poland; ‡ Doctoral School of Exact and Natural Sciences, Jagiellonian University in Kraków, Prof. S. Łojasiewicza 11, 30-348 Kraków, Poland

**Keywords:** metal−organic frameworks, click reactions, Diels−Alder, mechanochemistry, covalent modifications, adsorption, sensors, luminescence

## Abstract

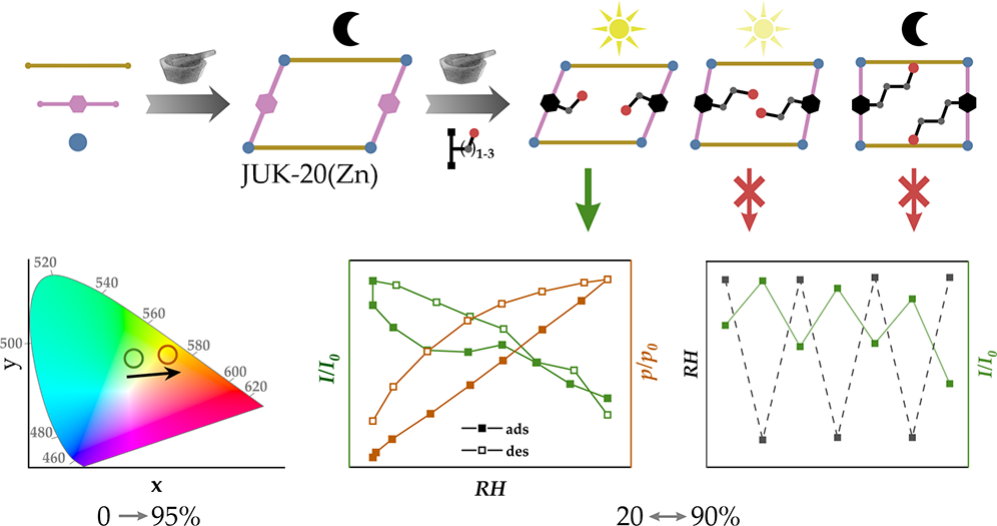

Covalent linker transformations in metal–organic frameworks (MOFs) enable their functionalization but often suffer from low conversions or require harsh conditions, including heating, corrosive reactants and solvents, or catalysts. In this work, using solvent-free mechanochemistry for the first time for such conversions, we demonstrate the systematic MOF pore modification with pendant hydroxyl groups and the resulting effects on the network rigidity, its luminescent properties, as well as adsorption of CO_2_ and vapors of methanol, ethanol, isopropanol, D_2_O, and H_2_O. A new zinc-based heterolinker MOF (JUK-20) containing both protic luminescent units and reactive tetrazine cores was used as a model and subjected to an inverse electron-demand Diels–Alder (iEDDA) click reaction with a series of dienophiles (x) of different lengths having OH groups. From the obtained series of JUK-20(Zn)-x MOFs, a flexible material capable of luminescent humidity sensing was identified, and the influence of water on the luminescence of the material was explained by analogy with the excited-state intramolecular proton transfer (ESIPT) model. In general, our results provide guidance for designing and tuning MOFs for luminescence-based detection using a stepwise synthetic approach.

## Introduction

Metal–organic frameworks (MOFs) are porous hybrid inorganic–organic materials that can be engineered for targeted applications taking advantage of reticular coordination chemistry with appropriate selection of metals and organic ligands,^1−3^ and at the supramolecular level through the use of motifs tailored to specific intermolecular interactions.^4^ Such functional MOFs, for example, selective adsorbents,^5^ catalysts,^6,7^ or sensors,^8,9^ are obtained by direct assembly of building blocks, possibly followed by final modification. Among the latter, covalent modifications of MOFs, although relatively rare, enable functionalization when direct incorporation of functional linkers is hindered by the presence of bulky or interacting groups.^10−14^ These modifications involve the formation or breaking of a covalent bond and are highly efficient, fast, and selective toward a designed reaction center. A promising group of covalent modifications are cycloadditions,^15−17^ including the inverse electron-demand Diels–Alder (iEDDA) reaction, which has recently been used to tune framework flexibility^18^ or enhance hydrophobicity.^19^ Most of the literature reports regarding MOFs and iEDDA processes, however, focus on the MOF reactivity and the modification itself.^20,21^

Both the direct synthesis of MOFs and their postsynthetic modifications are conventionally carried out using solvents (e.g., *N*,*N*-dimethylformamide) and heating. An alternative, more sustainable approach is offered by solvent-free mechanochemistry.^22,23^ With minimal or no solvent consumption, quantitative yields with zero byproducts, short reaction times, no solubility demands, and no heating, mechanochemical methods can meet most of the requirements of green chemistry.^24^ Compared to numerous examples of solvent-free *de novo* synthesis of MOFs (including well-known platforms: MOF-74, MOF-5, UiO-66, ZIF-8),^25−28^ the library of their postsynthetic mechanochemical modifications contains just a few complexation reactions,^29−31^ and to the best of our knowledge, no mechanochemical covalent modification has been reported. On the other hand, in the literature, there are several types of solvent-free covalent reactions involving organic compounds, sometimes never reported as in-solution variants.^32−35^ Successful mechanochemical protocols have also been described for metal-catalyzed reactions,^36^ for inorganic transformations involving main group elements,^37^ and in organometallic chemistry.^38^

In this work, we present the first covalent postsynthetic modification of a MOF by using mechanochemistry in parallel to an in-solution approach. As a model, we used a new zinc-based JUK-20 MOF containing a tetrazine core, a selection of OH-containing dienophiles (x) of various lengths, and the iEDDA click reaction. It is also noteworthy that the total synthesis of final MOFs is solvent-free, starting from linker precursors through the parent JUK-20(Zn) to the daughter materials JUK-20(Zn)-x. The dienophiles with OH groups were deliberately selected to induce hydrogen-bond interactions with alcohol adsorbates and linker carbonyl groups for potential control of framework rigidity and its response to adsorbates. In the literature, the OH groups inside the pores of MOFs, present either as nodal ligands or substituents in confined guest molecules, have recently been shown to significantly change host–guest interactions with adsorbates. As a result, higher uptakes and/or adsorption reversibility were achieved for H_2_S, SO_2_, CO_2_, or NH_3_ in such MOFs as MIL-53(Al)-TDC/-BDC,^39,40^ InOF-1,^41^ and MFM-300(Sc).^42,43^ Herein, we demonstrate a series of mechanochemically prepared JUK-20(Zn)-x MOFs with pendant OH groups, of varying structural flexibility and adsorption properties, including the material capable of luminescence-based humidity detection. The multistep synthesis, structure–adsorption–luminescence correlations, as well as the proposed mechanism of water detection are discussed.

## Results and Discussion

### Syntheses and Structures of the JUK-20(Zn)-x Series

**Figure 1:**
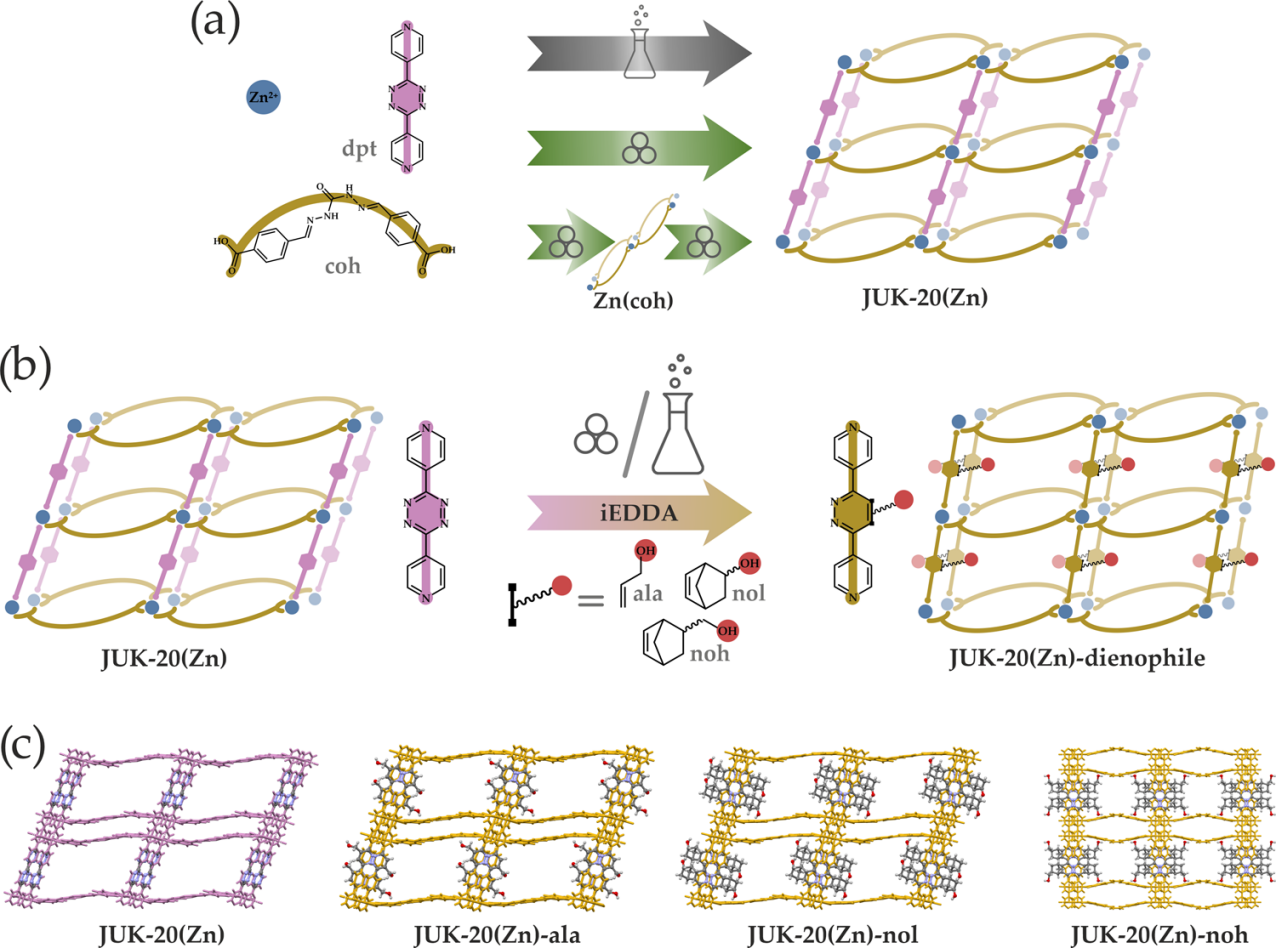
(a) Synthetic scheme leading to microporous JUK-20(Zn), including one-step synthesis in solution and one- or two-step mechanosynthesis. (b) iEDDA postsynthetic modification scheme, carried out for JUK-20(Zn). (c) Crystal structures of JUK-20-dienophile MOFs with tetrazine-derived linkers after iEDDA reactions (projections along the [001] direction for JUK-20(Zn), -ala, and -noh and along the [010] for JUK-20(Zn)-nol).

JUK-20(Zn) material was obtained by several alternative methods (Figure 1a; see the Supporting Information for details). First, heating of Zn(NO_3_)_2_·6H_2_O, coh, and dpt in DMF/MeOH (9:1 v/v), carried out at 80 °C for 48 h, led to a polycrystalline material {[Zn_2_(coh)_2_(dpt)_2_]·5DMF·3H_2_O}*_n_*, and its single crystals suitable for SC-XRD measurements were obtained by a slow diffusion method. A more sustainable approach was then developed as either a one-pot or a three-step mechanochemical variant (including coh ligand mechanosynthesis). JUK-20(Zn) is a 2D MOF that is isostructural to its cadmium-based counterpart JUK-20(Cd).^18^ The structure includes coordination layers with *sql* topology, which are held together by strong C=O···H–N hydrogen bonds between every second layer (structure scheme: Figure 1a). Thus, JUK-20(Zn) can also be viewed as a doubly interpenetrated 3D hybrid MOF–HOF polymer. The as-synthesized JUK-20(Zn) contains open one-dimensional diamond-shaped channels with large apertures of 9.6 × 15.9 Å^2^, propagating along the [001] direction. The accessible probe-occupiable pore volume of JUK-20(Zn), calculated using Zeo++ software^44^ with a probe radius of 1.86 Å, amounts to 44.3%.

In contrast to the previous studies of JUK-20(Cd), in which we presented the covalent incorporation of a series of dienophiles primarily differing in bulkiness, the aim of this work was to incorporate a hydroxyl group into JUK-20(Zn) using dienophiles of different lengths to control its flexibility and adsorption properties.

Three alcohols were chosen for this purpose: allyl alcohol (ala), 2-norbornenyl alcohol (nol), and (norbornenyl)methanol (noh) (Figure 1b) so that the hydroxyl group was separated from the reaction center by one, two, or three carbon atoms, respectively. The iEDDA click reactions involving the three dienophiles were carried out using polycrystalline samples and two approaches. In the first one, the reaction was performed in a DMF suspension with a small excess of a dienophile (see the Supporting Information for details). The high reactivity of dienophiles is related to the presence of an activating hydroxyl group and angular stress. Based on these features, the series of alcohols in order of increasing reactivity can be arranged (ala < noh < nol), which is consistent with the experimental observations. The progress of the reactions was monitored by UV–vis spectroscopy and other techniques (i.e., ^1^H NMR and IR spectroscopy, see the Supporting Information for details). In the second approach, each of the dienophiles quantitatively reacted with JUK-20(Zn) under solvent-free conditions to give a phase-pure JUK-20(Zn)-x product. To the best of our knowledge, this is the first example of a covalent postsynthetic modification of a MOF carried out mechanochemically. Several attempts have also been made to obtain JUK-20(Zn)-x materials *de novo*, using the preassembled dpt-dienophile ligands; however, no JUK-20(Zn)-x MOFs could be obtained. On the other hand, the modifications carried out on single crystals of JUK-20(Zn) (in suspension), despite full conversion to phase-pure products, led to disintegration of the crystallites due to high reaction rates. Although the global crystallinity of the samples was preserved, the materials were not useful for SC-XRD studies. In order to solve the crystal structures of the products, the isostructurality of the JUK-20(Cd) and (Zn) materials (see Figure S1) was used, and the same click modifications were carried out on the Cd material. Thus, the structures shown in Figure 1c are the result of isomorphous substitution for Zn ions in the JUK-20(Cd)-dienophile materials. For the materials obtained in the reactions with two shorter dienophiles (ala and nol), the structural parameters change only slightly as compared to the parent MOF material. The β-angle measure increases (from about 67.79 to 70.52 and 73.81°, respectively), which is related to the gradual decrease of a pillar-layer tilt due to the appearance of a substituent with increasing volume. Only the modification with the longest dienophile, noh, leads to a full reduction of the tilt (the crystal system becomes monoclinic). In the case of the modification with the ala dienophile, a half of its hydroxyl groups is too distant from the coh colinker to be involved in the formation of a stabilizing hydrogen bond, and the second half forms very weak hydrogen bonds (*d*(O_ala_···O_coh_) = 3.32 Å). For JUK-20(Zn)-nol, also only a half of hydroxyl groups is involved in hydrogen bonding with the coh ligand (*d*(O_nol_···O_coh_) = 2.99 Å). In contrast, for the JUK-20(Zn)-noh material, all hydroxyl groups form strong hydrogen bonds with the coh ligand (*d*(O_noh_···O_coh_) = 2.82 Å), serving as rigidifying anchors, which enable subnetwork crosslinking in the MOF and causing the largest structural transformation in the series discussed. In this way, the anchoring effect in JUK-20(Zn)-noh provides the highest stability for the JUK-20 platform. It is noteworthy that the proper length of the OH arm in a dienophile is essential to achieve this effect.

In general, the first visible symptom of the ongoing iEDDA reactions was the color change of the solids from pink to yellow due to the conversion of the 1,2,4,5-tetrazine moiety to 1,2-diazine (or dihydro-1,2-diazine). On this basis, solid-state UV–vis-NIR spectroscopy was used to monitor the completeness of the JUK-20(Zn) transformation. The presence of coh and dpt ligands in the initial MOF was also confirmed by infrared spectroscopy, and the disappearance of the characteristic band originating from the 1,2,4,5-tetrazine core was observed in the reaction products. Additionally, for digested MOFs in deuterated D_2_SO_4_/DMSO-*d*_6_ mixture, ^1^H NMR spectroscopy was used to demonstrate the structure of all ligands. The composition of each material and the amount of guest molecules were examined by elemental analysis and thermogravimetry (for details, see the Supporting Information, page S4 and Figures S3 and S4).

To estimate the environmental impact of both in-solution and solvent-free syntheses, we performed a comparative analysis of selected “green” performance indicators (green metrics).^45^ The calculated parameters include the relative amount of waste (E-factor), the process mass intensity (PMI), defined as the relative mass of the reaction mixture necessary to obtain a given amount of product, and the reaction mass efficiency (RME), which is a measure of the atomic efficiency of the reactions carried out, taking into account the masses of the substrates used in reality (see the Supporting Information). In addition, we calculated the energy consumption per kilogram of the product and the percentage of energy saved by choosing the mechanochemical approach. The values of the calculated factors are collected in Table 1. The comparison of the final step of synthesis (iEDDA modification) is based on the modification involving dienophile ala.

**Table 1:** Comparison of Conventional In-Solution and Mechanochemical Syntheses of the coh Ligand, JUK-20(Zn), and JUK-20(Zn)-ala in Terms of Green Metrics Factors

		yield (%)	E-factor	process mass intensity (PMI)	reaction mass efficiency (RME, %)	energy consumption (kWh·kg^–1^)	energy saved (in mechanochemical approach, %)
coh ligand synthesis	in-solution	81	60.7	61.7	73.7	296	20.7
	mechanochemical	>95	1.64	2.64	90.7	235	
JUK-20(Zn) synthesis	in-solution	68	159	160	0.684	109 000	99.5
	mechanochemical	>95	0.828	1.83	54.7	579	
JUK-20(Zn)-ala synthesis	in-solution	>95	29.5	30.5	57.1	12 400	89.8
	mechanochemical	>95	11.5	12.5	7.98	1 270	

By analyzing the data in Table 1, a general conclusion can be drawn that for each of the synthesis steps, the mechanochemical variant is much more favorable from an ecological and economic point of view. The smallest differences concern the synthesis of the ligand, due to the relatively short duration of the synthesis in solution and its high yield. The most significant differences are in the synthesis of the JUK-20(Zn) MOF, where mechanosynthesis allows for an almost 200-fold reduction in the amount of waste produced and results in energy savings of 99.5%. The use of mechanosynthesis for postsynthetic modification is particularly favorable from the point of view of energy savings (89.8% change); however, considering the amount of waste generated and the reaction mass efficiency, the overall advantage is not so high, mainly due to the need to use a large excess of the less reactive ala dienophile for mechanosynthesis.

### Porosity and Structural Flexibility of JUK-20(Zn)-x

**Figure 2:**
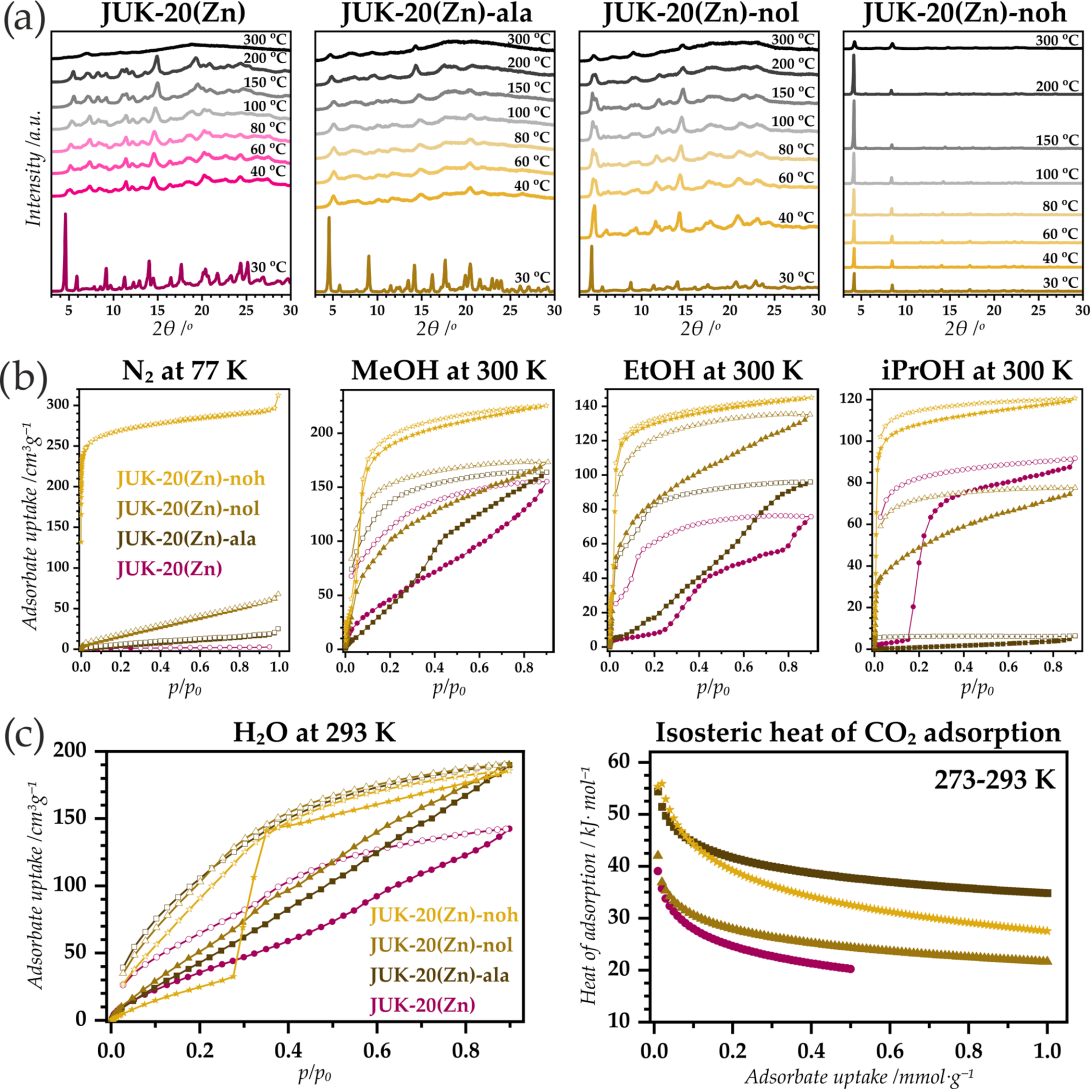
(a) VT-PXRD patterns of the JUK-20(Zn)-x series. (b) Physisorption isotherms of nitrogen (at 77 K), methanol, ethanol, and 2-propanol (at 300 K each) measured for the JUK-20(Zn)-x series. (c) Water and CO_2_ physisorption for the JUK-20(Zn)-x series: experimental isotherms for water (at 293 K) and isosteric heat of adsorption profiles for CO_2_ (based on the measurements at 273, 283, and 293 K).

In the literature, introducing dienophiles with increasing bulkiness to the JUK-20(Cd)-x series led to a stepwise rigidification of the initial framework.^18^ In this cadmium-based family, all partially rigidified materials occurred in a monoclinic system, leading to a hypothesis that, in the case of the JUK-20(Zn)-ala and nol materials, despite the presence of a hydroxyl group, their flexibility, and consequent properties, may be retained. To prove this hypothesis, the following studies have been carried out. The first investigated rationale was the behavior of the entire JUK-20(Zn)-x series after the removal of guest molecules and subsequent resolvation (Figure S5). Only for JUK-20(Zn)-noh, the material fully retained crystallinity after activation; the other three materials were partially amorphized, but their crystallinity was completely recovered after resolvation. This behavior was further explored by examining the X-ray diffraction of the materials as a function of temperature (Figure 2a). The JUK-20(Zn) material behaves similarly to its cadmium analogue: at low temperatures, the crystallinity is partially lost, and the remaining broadened Bragg reflections shift toward higher 2θ angles. Analogous behavior is demonstrated by JUK-20(Zn)-ala. For the nol-modified material at temperatures within 60–200 °C, two phases are observed, with a dominant contribution of the structure with the original unit cell parameters. This may indicate that the material is rigidified to some extent only, as compared to fully rigid JUK-20(Zn)-noh, whose hydroxyl groups play the anchoring role by crosslinking the interpenetrating subnetworks of the MOF.

In general, flexible MOF materials may exist in closed (cp) or open (op) phases dependent on conditions. To verify the state of the JUK-20(Zn)-x frameworks after activation, nitrogen physisorption was examined (Figure 2b). Only for JUK-20(Zn)-noh, type I isotherm was recorded, and the pore volume calculated using the Gurvich rule is equal to 0.46 cm^3^/g, which is slightly higher than the virtual porosity estimated for the structural model (0.36 cm^3^/g). For the other materials, the experimental pore volume available for nitrogen is much smaller than the virtual porosity and reaches a maximum of 28% of the potential free space for JUK-20(Zn)-nol (see Table S1 and Figure S6). Using the BET model, the specific surface area values for the studied series of materials were estimated to be 5, 28, 87, and 1051 m^2^/g, respectively. From these observations, it can be concluded that JUK-20(Zn) and JUK-20(Zn)-ala are closed pore phases (cp) after activation and JUK-20(Zn)-noh has fully open pores (op), while JUK-20(Zn)-nol shows very low porosity and is open to a very slight degree. The exploration of porosity of the synthesized materials was extended for other adsorbates as well. In a previous study, GCMC modeling of the adsorption isotherms of methanol was carried out for the JUK-20(Cd)-noh material. The analysis showed that in the first stage, the adsorbate is located in hydrophobic areas of the pores, and only upon their filling, a stepwise adsorption associated with condensation in the central part of the pore occurs. With this fact in mind, the sorption properties for the series of JUK-20(Zn)-x materials were investigated by using three alcohols with increasing hydrophobicity: methanol, ethanol, and isopropanol (Figure 2b). In the case of the rigidified material, JUK-20(Zn)-noh, the curves similar in shape to the Type I isotherms were observed for all alcohols; however, with an apparent step occurring at partial pressures the lower the more hydrophobic the adsorbate, confirming the validity of the model described earlier (cf. Figure S7). In the case of the remaining materials, methanol appears to be a universal adsorbate that fills a moderate part of the pores (50–90%), while ethanol fills the free spaces of the JUK-20(Zn)-nol material completely (for the other materials, only 39 and 61%, respectively). Isopropanol, despite its greater hydrophobicity, is a larger molecule and is adsorbed by nonrigidified materials only to a small extent (5–57% of the available free space). The analysis presented here confirms that except for JUK-20(Zn)-noh, the studied materials are flexible, the removal of guest molecules results in their transformation to the cp phases, and the appropriate adsorbates are, to some extent, capable of their transformation to the op phases. The transition in most cases is not abrupt, as for typical materials exhibiting breathing behavior, but continuous, a near linear increase in the adsorbed volume is observed with an increase of the partial pressure of the adsorbate.

The adsorbate that demonstrates even higher polarity than methanol is water. JUK-20(Zn)-noh exhibits a particularly interesting behavior toward water vapor (Figure 2c). Although it is fully rigidified, the maximum amount adsorbed (at *p*/*p*_0_ = 0.90) is only ∼37% of the available void volume. The explanation of this phenomenon can be based on the modeled stages of methanol sorption for the cadmium-based counterpart.^18^ The key stage is the filling of hydrophobic pore areas, manifested for alcohols by a step on the adsorption curve. In the case of the water adsorption isotherm, this step does not appear until *p*/*p*_0_ = 0.30, which may indicate a high activation barrier. Further filling of the pores is also not complete, and it occurs mainly at the pore walls, where hydrogen bond donors and acceptors are present. Based on this reasoning, important conclusions can be drawn. The sorption capacity at *p*/*p*_0_ = 0.90 for each material can be equated with the number of hydrogen bond donors and acceptors available. Consistently, the starting material JUK-20(Zn) is the only one that adsorbs less water than the other materials. In terms of the number of adsorbate molecules per asymmetric unit, this material takes up from 3 to 3.75 molecules less since it is the only one without hydroxyl groups in its structure. Another important feature of water adsorption isotherms is their shape. For all MOFs, the recorded curves differ significantly from typical isotherm shapes, and in the case of JUK-20(Zn)-ala and JUK-20(Zn)-nol, an almost linear increase in the amount of adsorbed water is observed with an increase in its partial pressure. This effect is explained by a combination of two features of these materials.

First, both are highly flexible, so the cp phases are exposed to the adsorbate. In the initial stages of adsorption, only a small amount of adsorbate can fill the small volume of the remaining pores, but with each subsequent portion, a gradual transformation from the cp phase to a partially open phase takes place. Second, the presence of hydrophobic areas and polar pore walls is important, as described for the rigidified JUK-20(Zn)-noh.

Although the adsorption capacity of water vapor gave a self-evident indication of the presence of additional hydrogen bond donors and acceptors in the studied materials, to investigate the presence of free (nonbonded) hydrogen bond donors, it is necessary to use different adsorbates. For this purpose, carbon dioxide was chosen, and its isosteric heat of adsorption was determined by physisorption measurements in the range of 273–293 K (Figures 2c and S8). The material for which the highest heat of adsorption was observed for higher adsorption capacities is JUK-20(Zn)-ala, indicating the presence of free hydrogen bond donors. This result coincides with the structural model, which indicates that the hydroxyl groups in this material are not hydrogen-bonded, and the cp phase under study must also have these groups in the free state. For JUK-20(Zn)-nol, the heat of adsorption is relatively high only in the initial stage of adsorption. This indicates the absence of additional functional groups in the starting material and the interaction of the OH groups in JUK-20(Zn)-nol via hydrogen bonds in the cp phase under study. The most rigidified JUK-20(Zn)-noh shows the highest heat of adsorption for the smallest adsorption capacities due to the direct accessibility of the NH groups in the coh ligand and the norbornene pockets of the op phase, while for larger adsorption capacities, the heat of adsorption decreases faster than for JUK-20(Zn)-ala, due to the smaller amount of available hydrogen bond donors.

The detailed mechanism of CO_2_ adsorption was proposed for the cadmium-based analogue JUK-20-noh based on grand canonical Monte Carlo simulations. It was shown that the initial step involved a preferable location of the adsorbate inside the hydrophobic norbornene pockets.

To further explore the porous nature of the materials studied, we investigated their behavior with respect to D_2_O vapor as an adsorbate. Although the separation of water isotopomers has been a long-standing problem, it remains a challenge to perform in an efficient and sustainable manner. The chemistry of MOFs enables unique separation pathways for different substances, and an interesting report has recently been published in this area.^46^ In the case of the JUK-20(Zn) family of materials, the presence of hydrophilically decorated pores combined with their structural flexibility has led to the observation of a different behavior between H_2_O and D_2_O (see Figure S9). Deuterated water exhibits a higher affinity for hydrophilic pockets as a result of a higher hydrogen bond strength.^47^ Since water adsorption is a very dynamic process driven by successive portions of the adsorbate, the greatest differences appear for the most flexible materials, JUK-20(Zn) and JUK-20(Zn)-ala, which adsorb about 60 and 30% more D_2_O than H_2_O, respectively, over most of the pressure range. The rigid JUK-20(Zn)-noh shows significantly higher affinity for D_2_O only at very low partial pressures (below *p*/*p*_0_ = 0.02); for the rest of the pressure range, the difference is quite low (about 13%). The most complex behavior is demonstrated by JUK-20(Zn)-nol, for which there is no difference between these adsorbates until *p*/*p*_0_ = 0.30, where a step in the D_2_O adsorption curve appears, and D_2_O uptake becomes about 80% larger than H_2_O. For higher partial pressures, the preference for D_2_O decreases to 27%.

### Photoluminescence and Humidity Detection

Whereas the luminescence of the dpt ligand has been described in the literature in solution,^48^ there are no reports on such measurements carried out for a solid. Our research revealed that the dpt ligand, both at room temperature and at 77 K, exhibits emission too weak to be recorded with the instrument used; however, the coh ligand exhibits strong emission dependent on temperature (for λ_exc_ = 365 nm, intense blue emission with a band maximum at ca. 420 nm at 77 K, and green emission with a band maximum at ca. 496 nm at 293 K; see Figure S10). Consequently, intense emission could be expected for the JUK-20(Zn) material that contains both ligands, at least originating from the coh ligand. However, both the as-synthesized (op, with guest molecules) and the activated samples (cp, guest-free) do not show even trace emission upon excitation in the 300–550 nm range. The first reason for that is a direct quenching of coh emission by dpt (see coh emission and dpt reflectance spectra, Figure S10e). On the other hand, the hypothetical emission from the excited dpt ligand is quenched by vibrations of the backbone and (in the case of the as-synthesized sample) the guest molecules.

The iEDDA-modified materials were examined for luminescence as both as-synthesized and activated phases. Of the three materials tested, only JUK-20(Zn)-ala showed intense green light emission in both phases. The luminescence of the JUK-20(Zn)-nol material was very weak and only recordable for the activated phase (cp). JUK-20(Zn)-noh did not exhibit luminescence in any of the phases tested (Figure S11).

Considering the structural features of the frameworks of the studied materials, it can be hypothesized that in order to observe luminescence, both the flexibility of the material and the presence of an OH group are necessary. However, this is not a sufficient requirement. It is worth noting that for all MOFs after the iEDDA reaction, their 1,2,4,5-tetrazine system vanishes, and thus the direct cause of the luminescence quenching of the coh ligand disappears. In the case of JUK-20(Zn)-noh, luminescence is not observed, which is caused by the formation of strong hydrogen bonds between the dpt-noh and coh ligands. This direct-contact interaction vibrationally quenches the luminescence originating from the coh ligand. On the other hand, weak luminescence of the JUK-20(Zn)-nol material indicates that after activation to the cp phase, most of the OH groups are also in contact with the coh ligands through hydrogen bonds. Only in the case of the shortest of the ligands, dpt-ala, even in the activated (cp) phase, there is no contact with the coh ligand to quench its luminescence. To confirm that the coh ligand is the source of emission and that the presence of the dpt-ala ligand is crucial, we investigated the luminescence properties of the Zn(coh), dpt-ala, and JUK-20(Zn)-eve compounds as references (eve stands for ethyl-vinyl ether, and its iEDDA reaction product is the dpt-eve ligand differing from dpt-ala only by the absence of the CH_2_OH group). The Zn(coh) material (i.e., mechanochemically obtained zinc-based coordination polymer with coh ligands only, see Figure 1a) also shows intense bluish emission at room temperature (emission band maximum at 468 nm for λ_exc_ = 420 nm), while neither dpt-ala nor JUK-20(Zn)-eve (as the as-synthesized or activated phase) shows a signal of sufficient intensity to be registerable. This finding confirms the key role of hydroxyl groups in the observability of the coh ligand emission (Table S2).

**Figure 3:**
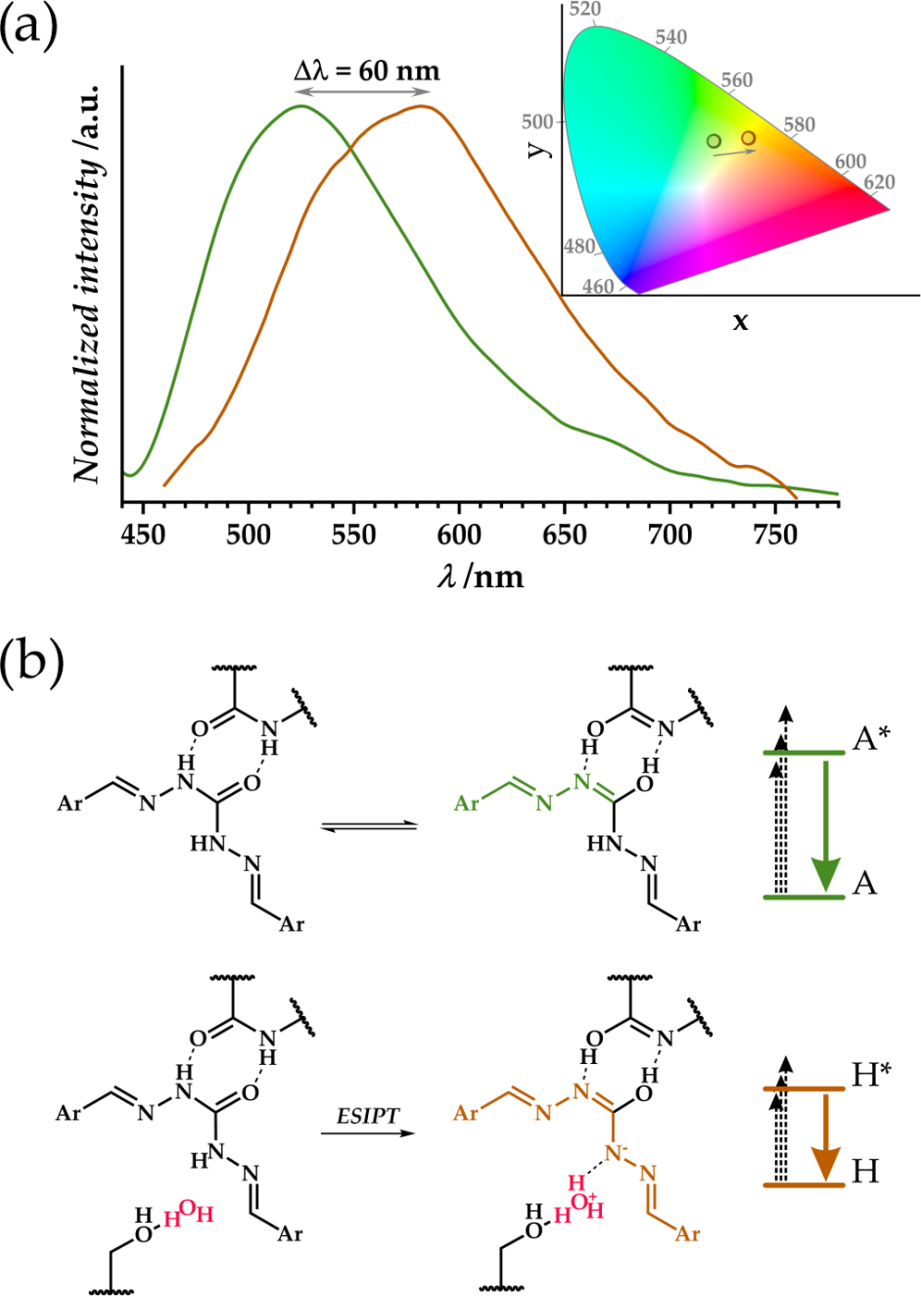
(a) Normalized luminescence spectra for anhydrous (A, green) and hydrated (H, brown) phases of JUK-20(Zn)-ala. Chromatic diagram with desolvation-induced emission shift visualized. (b) Formation of the enol tautomer of the coh ligand with extended delocalization in a desolvated phase (top) and the analogous scheme for a hydrated phase (bottom), with a proton transfer to an adsorbed water molecule.

As presented in the previous paragraph, the JUK-20(Zn)-ala material shows an unusual elastic behavior toward water as an adsorbate, based on which a linear change in the luminescence parameters of this material can be expected with varying relative humidity. Figure 3a illustrates the change in emission spectra upon transition from the activated JUK-20(Zn)-ala to the material conditioned under RH = 94% (excitation at λ = 440 nm). A significant bathochromic emission shift Δλ = 60 nm is observed, which is associated with a transformation of the coh ligand. The mechanism of the observed effect is proposed in Figure 3b, which explains the crucial presence of the hydroxyl group and the water molecule in the excited state upon proton transfer. This transfer takes place within the supramolecular system and is therefore a variant of the ESIPT process, resulting in a significant extension of the conjugated system and a reduction in the energy difference between the HOMO–LUMO levels. By analogy with the JUK-20(Cd)-noh material,^18^ the first site where water molecules are adsorbed is the pocket between the coh ligand and the central part of the modified dpt ligand. In the case analyzed, a water molecule at this site can form strong hydrogen bonds with both the coh and the dpt-ala ligands, as shown in Figure 3b. Vacuum desolvation of the sample at this stage leads to a return to the original state, but a nitrogen-flow desolvation results in an increase in emission intensity only, with no shift in the emission wavelength. Compared with consecutive water adsorption isotherms, it should be noted that some part of the adsorbate remains in the pores at these conditions (Figure S12), and luminescence studies indicate that it must be guest molecules directly affecting the significant emission red shift.

**Figure 4:**
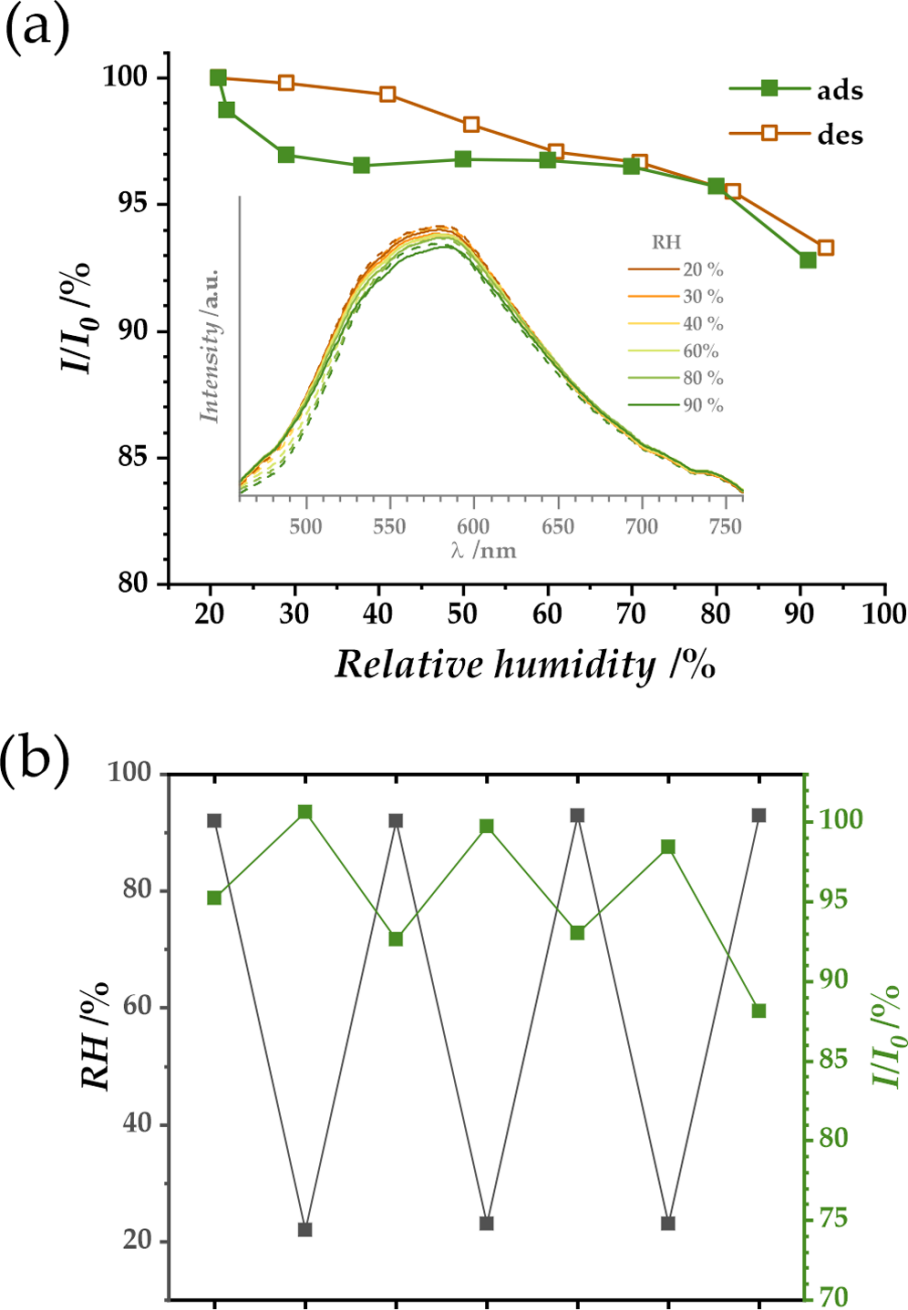
(a) Humidity-dependent emission isotherm (for λ_em_ = 582 nm, at 293 K) for JUK-20(Zn)-ala with corresponding emission spectra for selected points of adsorption (straight line) and desorption (dashed line) shown as an inset. (b) Reproducibility of the sensory effect, shown for three consecutive cycles of 90–20–90% RH.

The JUK-20(Zn)-ala material exhibits a luminescence response to a change in humidity also in the nonactivated phase. In this case, a different effect is observed - a decrease in emission intensity with increasing humidity, with no change in emitted light energy. Water molecules adsorb in the pores around the organic luminophore; their presence increases the number of OH vibrational levels, whose overtones resonantly overlap with the emission level of the ligand, thus quenching its emission.^49^ Therefore, the material behaves as a humidity sensor, since a change in the amount of water in the pores directly affects the emission intensity of the coh ligand. Figure 4 shows the emission isotherm as a function of relative humidity, measured for the first adsorption cycle in the range RH = 20–90% (for the excitation isotherm, see Figure S12). The photograph of a planar sensor, including both activated and humidified polycrystalline samples of JUK-20(Zn)-ala under UV light positioned on a glass support, is shown in Figure S13. The reproducibility of the result obtained was tested for three consecutive cycles of 90–20–90% RH (Figure 4b). The presence of hysteresis in the water vapor adsorption and light emission isotherms confirms the direct relationship between the amount of water in the pores and the emission intensity. The effect described is more subtle than in the case of the first exposure of the activated material to moisture but allows its use for humidity detection without the need for vacuum activation before each measurement. A consequence of the flexibility of the material is a slight decrease in the sensor response to a given stimulus over subsequent cycles.

Regardless of the fundamental factors for moisture sensors, such as the choice of transduction technique and integration of materials into the devices,^50^ an obvious limitation of the presented system is the poor luminescence quenching/enhancement effect of humidification and drying. From a materials perspective, however, MOFs offer ample opportunities to improve their performance, such as through careful selection of building blocks, including luminescent nodes or linkers, compared, for example, to zeolites, whose films have also been used as moisture sensors with capacitive or current response.^51,52^

## Conclusions

In conclusion, we have presented the first mechanochemical covalent modification of a MOF by using inverse electron-demand Diels–Alder (iEDDA) click reaction. A series of systematic solvent-free modifications of a tetrazine-based model MOF using dienophiles of different lengths with hydroxyl groups allowed us to influence both the structural rigidity as well as the adsorption and luminescent properties of the material. A dienophile with the appropriate OH arm length was tailored to exhibit intraframework hydrogen-bonding interaction, and the modified MOF showed combined flexibility with a luminescent sensory response to water vapor. The presented mechanochemical, postsynthetic functionalization approach may be extended to other flexible metal–organic frameworks containing a tetrazine ring, and the described mechanism for total material activation should be used to enhance the sensory effect. Careful design of a new luminescent linker may enable a strong ESIPT effect without the necessity of vacuum activation of the material while maintaining its structural flexibility and a narrow emission hysteresis.

## Experimental Section

### Materials

All reagents and solvents, unless otherwise noted, were purchased from commercial sources and used without further purification. 3,6-Di(pyridin-4-yl)-1,2,4,5-tetrazine (dpt) and bis(4-formylbenzoic acid) carbohydrazone (coh) were synthesized using a literature method.^18^ The coh ligand was furthermore obtained mechanochemically (see the Supporting Information for details). (Bicyclo[2.2.1]hept-5-en-2-yl)methanol (noh) and bicyclo[2.2.1]hept-5-en-2-ol (nol) were synthesized by literature methods.^18,53^ Derivatives of the dpt ligand (i.e., dpt-ala, dpt-nol, dpt-noh) were synthesized by iEDDA reaction with a corresponding dienophile (see the Supporting Information for details).

### MOF Syntheses

Single crystals of JUK-20(Zn) were obtained by a slow diffusion method. Bulk samples were prepared in closed vials using a mixture of *N*,*N*-dimethylformamide (DMF) and methanol (MeOH) at 80 °C and alternatively by mechanochemical reaction between zinc(II) acetate and ligands in two variants. The JUK-20(Zn) iEDDA reactions with all dienophiles were conducted by using solids suspended in DMF or alternatively by grinding the precursor material with appropriate dienophile, without solvent addition. The reaction conditions were established by monitoring UV–vis and nuclear magnetic resonance (NMR) spectra of the products. Additional control syntheses involving the pre-assembly modified dpt linkers (dpt-ala, dpt-nol, dpt-noh) did not lead to JUK-20(Zn)-dienophile products (see the Supporting Information for details).

### Physisorption and Photoluminescence Measurements

The adsorption measurements for N_2_, CO_2_, water (H_2_O and D_2_O), and alcohol vapors were performed at 77, 273–293, 293, and 300 K, respectively. The virtual porosity of JUK-20-x was analyzed using Zeo++ calculations of the framework models based on the crystal structures (Table S1).^44^ The carbon dioxide heat of adsorption was determined by fitting a Freundlich model for carbon dioxide adsorption isotherms at three temperatures: 273, 283, and 293 K. Solid-state photoluminescent characterization for all reported compounds was performed using an FS5 spectrofluorometer (Edinburgh Instruments) equipped with a Xe arc lamp (150 W, excitation spectra) serving as an excitation source and a Hamamatsu photomultiplier of the R928P type as a detector. Humidity-dependent measurements were performed in situ using a home-made setup employing HG-100 RH humidity generator (L&C Science and Technology).

## Supporting Information Available

Details of synthetic procedures, sample handling, physical measurements, and computational studies; additional IR, PXRD, TGA, UV–vis, NMR, adsorption, and spectrofluorimetric data; pictures of solids obtained during total mechanosynthesis; and structural analysis details (PDF)
