# Supplementary material for: Covalent Modification by Click Mechanochemistry: Systematic Installation of Pendant OH Groups in a MOF for Rigidity Control and Luminescence-Based Water Detection
Source: ACS Appl Mater Interfaces. 2023 May 19;15(21):25661–70. doi: 10.1021/acsami.3c00788 (PMC10236426; doi:10.1021/acsami.3c00788)
Supplement: Supplementary file 1 — am3c00788_si_001.pdf [file am3c00788_si_001.pdf]

SUPPORTING INFORMATION

Covalent modification by click mechanochemistry: systematic installation of pendant OH groups in a MOF for rigidity control and luminescence-based water detection

Damian Jędrzejowski[1,2], Michał Ryndak[1], Jakub J. Zakrzewski[1,2], Maciej Hodorowicz[1], Szymon Chorazy[1] and Dariusz Matoga[1,]*

[1] Faculty of Chemistry, Jagiellonian University in Kraków, Gronostajowa 2, 30–387 Kraków, Poland

[2] Doctoral School of Exact and Natural Sciences, Jagiellonian University in Kraków, prof. S. Łojasiewicza 11, 30-348, Kraków, Poland

e-mail: dariusz.matoga@uj.edu.pl

Contents:

Synthetic procedures 3
Details of mechanosyntheses and physical measurements 5
Calculations of green metrics factors 6
Figures and Tables 7
Figure S1. Comparison of powder XRD patterns measured for JUK-20(Zn)-x materials and their cadmium analogues 7
Figure S2. Characterization of JUK-20(Zn) 8
Figure S3. Characterization of the JUK-20(Zn)-dienophile series. 9
Figure S4. ^{1}H NMR spectra for JUK-20(Zn)-dienophile networks 10
Figure S5. Desolvation-resolvation of solid JUK-20(Zn) 11
Table S1. Structural analysis of JUK-20(M)-x series models determined by Zeo++ simulations and nitrogen adsorption measurements. 12
Figure S6. Void volume occupied by different adsorbates in the JUK-20(Zn)-x series 12
Figure S7. Adsorption and desorption isotherms for materials studied, measured for different adsorbates 13
Figure S8. CO_2 heat of adsorption studies 14
Figure S9. D_2O adsorption isotherms 15
Figure S10. Emission and excitation spectra for the coh ligand measured at 293 K and 77 K. Normalized emission spectra for the coh ligand measured 293 K and 77 K at different excitation wavelengths. Comparison of the coh ligand emission spectrum and reflectance spectrum of the dpt ligand 16
Figure S11. Excitation and emission spectra for activated and as-synthesized JUK-20(Zn)-ala material and for activated JUK-20(Zn)-nol material 17
Table S2. Comparison of luminescence parameters for materials studied 18
Figure S12. Consecutive cycles of JUK-20(Zn)-ala water vapor sorption. Excitation isotherm for JUK-20(Zn)-ala in varying humidity. Emission spectra for selected points of adsorption and desorption 18
Table S3. Optical images (under sunlight and UV light) of solid products obtained during total mechanosynthesis of JUK-20(Zn)-ala. 19
Figure S13. The photograph of a planar sensor including both activated and humidified polycrystalline samples of JUK-20(Zn)-ala under sunlight (a) and UV light (b) positioned on a glass support. 20
Structural analysis details 21
References 25

Synthetic procedures

All chemicals and solvents (of analytical grade), unless otherwise noted, were purchased from commercial sources (Merck, Fluorochem, Avantor) and were used without further purification.

Bis(4-formylbenzoic acid) carbohydrazone (coh):

A mixture of 60 mg (0.40 mmol) of 4-formylbenzoic acid and 18 mg (0.20 mmol) of carbohydrazide was placed together in 10 mL agate grinding jar with 4 agate balls (d = 10 mm). Then 100 μL of ethanol (η = 1.28 μL/mg) was added and the mixture was ground in a mixing mill with 15 Hz frequency for 20 minutes. The yellowish solid obtained was used without further purification. The lack of substrates in the reaction mixture was confirmed by thin-layer chromatography, FT-IR and ^{1}H NMR spectroscopy. By changing the volume of the grinding jar, this synthesis was scaled up by a factor of 20. ^{1}H NMR (600 MHz, DMSO-d_6): δ = 13.06 (br s, 2H), 10.96 (s, 2H), 8.24 (br s, 2H), 7.98 (d, J = 7.2 Hz, 4 H), 7.86 (d, J = 7.2 Hz, 4 H) ppm.

3,6-di(pyridin-4-yl)-1,2,4,5-tetrazine (dpt), (bicyclo[2.2.1]hept-5-en-2-yl)methanol (noh) were synthesized using methods previously described.[1] Coh ligand synthesis was scaled up by the factor of 20.

Bicyclo[2.2.1]hept-5-en-2-ol (5-norbornen-2-ol, nol)[2]:

A Teflon-lined stainless steel autoclave was filled with vinyl acetate (55 g, 639 mmol), dicyclopentadiene (14.1 g, 107 mmol), hydroquinone (22 mg, 0.20 mmol) and toluene (18 mL). The reaction mixture was heated to 180°C for 12 h. An excess of vinyl acetate and toluene was removed under vacuum, and the residual yellow oily liquid was purified by vacuum distillation. 19.6g (61% yield) of 5-norbornen-2-yl acetate was collected at 118-125°C under 10 mm Hg.

8.15g (53.6 mmol) of 5-norbornen-2-yl acetate, 0.58 mL (10.1 mmol) of a 25% (m/m) solution of sodium methoxide in methanol and 74 mL of methanol were placed in an Erlenmeyer flask. The mixture was stirred overnight. 10 mL of distilled water was added to the mixture followed by concentration under reduced pressure at 50°C to ca. 20 mL. The water phase was extracted with three 30 mL portions of diethyl ether, organic phase was washed with brine, dried over anhydrous sodium sulphate, and concentrated to ca. 10 mL under reduced pressure. The solid product was collected by vacuum resublimation and was identified as mixture of 5-norbornen-2-ol enantiomers (74% endo, 26% exo).

^{1}H NMR (600 MHz, $CDCl_3$), *endo*: δ = 6.44 (dd, *J* = 5.7, 3.1 Hz, 1H), 6.06 (dd, *J* = 5.7, 2.9 Hz, 1H), 4.47 (dt, *J* = 8.1, 3.2 Hz, 1H), 2.99 (s, 1H), 2.81 (s, 1H), 2.09 (ddd, *J* = 12.1, 8.1, 3.8 Hz, 1H), 1.47 (ddt, *J* = 8.5, 3.8, 2.0 Hz, 1H), 1.28 (d, *J* = 9.2 Hz, 1H), 0.77 – 0.73 (dt, *J* = 12.4, 3.1 Hz, 1H) ppm.

exo: δ = 6.16 (dd, *J* = 5.7, 2.9 Hz, 1H), 5.94 (dd, *J* = 5.7, 3.2 Hz, 1H), 3.89 (d, *J* = 6.8 Hz, 1H), 2.81 (s, 1H), 2.70 (s, 1H), 1.72 (d, *J* = 8.5 Hz, 1H), 1.64 (ddd, *J* = 12.1, 6.8, 2.6 Hz, 1H), 1.57 – 1.54 (m, 1H), 1.27 – 1.24 (m, 1H), ppm.

^{13}C NMR (150 MHz, $CDCl_3$), *endo*: δ = 140.6, 131.0, 72.4, 48.4, 48.2, 43.0, 37.9 ppm.

exo: 140.4, 133.5, 72.7, 50.3, 45.6, 40.8, 37.3 ppm. NMR was in accordance with the literature data.[3]

FT-IR (ATR, cm^{-1}, selected peaks): 3338 br s, 3064 w, 2970 vs, 2928 s, 2867 s, 1443 w, 1340 m, 1119 m, 1048 s, 1011 m, 722 s.

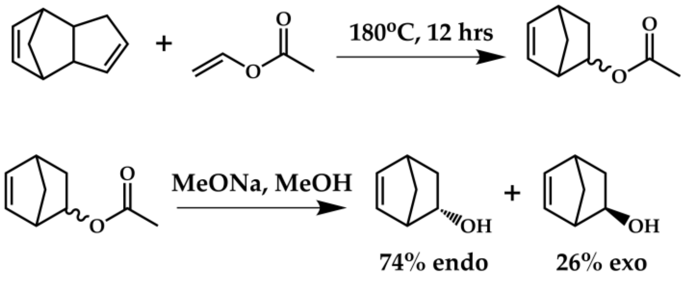

Synthesis of JUK-20(Zn), {$[Zn_2(coh)_2(dpt)_2]$·5DMF·3H_2O}$_n$:

446 mg (1.50 mmol) of zinc(II) nitrate hexahydrate, 534 mg (1.50 mmol) of coh ligand and 354 mg (1.50 mmol) of dpt ligand were dissolved in a mixture of 135 mL of N,N-dimethylformamide and 15 mL of methanol, distributed between 20 mL vials and heated to 80°C. After two days, red crystals of JUK-20(Zn) was filtered, washed with fresh N,N-dimethylformamide and air dried (881 mg, 68% yield). Attempts to scale up the reaction lead to contamination of the material with by-products. Elemental composition was determined for a desolvated sample. Calculated for $C_{58}H_{35}Zn_2N_{20}O_{17.5}$ ({$[Zn_2(coh)_2(dpt)_2]$·7.5H_2O·}$_n$): C 48.28%; H 3.85%; N 19.42%. Found: C 48.25%, H 3.43%, N 19.60%.

Growing single crystals of JUK-20(Zn):

22.3 mg (75.0 µmol) of zinc(II) nitrate hexahydrate was dissolved in 5 mL of methanol (solution A). 26.7 mg (75.0 µmol) of coh and 17.7 mg (75.0 µmol) of dpt were dissolved in 5 mL of N,N-dimethylformamide (solution B). Solution B was placed in a glass tube followed by careful addition of buffer MeOH/DMF (1:1 *v/v*) solution. On top of this, solution A was carefully layered and tube was sealed with parafilm. Red single crystals of JUK-20(Zn) appeared on the tube walls after approx. 7-10 days.

Mechanosynthesis of JUK-20(Zn):

Variant 1: 22.0 mg (0.10 mmol) of zinc(II) acetate dihydrate, 35.6 mg (0.10 mmol) of coh ligand and 23.6 mg (0.10 mmol) of dpt ligand were placed together in a 10 mL agate grinding jar with 10 agate balls (d = 7 mm). Then 81.2 µL of N,N-dimethylformamide (η = 1.00 µL/mg) was added and the mixture was ground in a mixing mill with 20 Hz frequency for 30 minutes. The pink solid obtained was identified as phase-pure JUK-20(Zn) (see Fig. S1). By changing the volume of the grinding jar, this synthesis was scaled up by a factor of 20.

Variant 2: 22.0 mg (0.10 mmol) of zinc(II) acetate dihydrate and 35.6 mg (0.10 mmol) of coh ligand were placed together in a 10 mL agate grinding jar with 10 agate balls (d = 7 mm). Then 28.8 µL of N,N-dimethylformamide (η = 0.50 µL/mg) and 28.8 µL of water (η = 0.50 µL/mg) were added and the mixture was ground in a mixing mill with a 20 Hz frequency for 30 minutes. The white solid obtained was identified as a Zn(coh) polymer (see Fig. S1). At the following stage of synthesis, 23.6 mg (0.10 mmol) of dpt ligand was added to Zn(coh) followed by 81.2 µL of N,N-dimethylformamide (η = 1.00 µL/mg) and the mixture was ground in a mixing mill with 20 Hz frequency for 30 minutes. The pink solid obtained was identified as phase-pure JUK-20(Zn) (see Fig. S1).

Reactions of dpt with dienophiles:

23.6 mg (100 µmol) of dpt was dissolved in 1 mL of dimethylsulfoxide and 500 µmol of appropriate dienophile was added to a vigorously stirred solution at room temperature. After some time (different for various dienophiles) the change of color of the solution from pink to yellow / pale-yellow and gas evolution was observed. TLC and UV-vis spectra monitoring allowed to determine the reaction completion. Dpt-dienophile ligands were then isolated by addition of a nonpolar solvent (diethyl ether) and used for further synthetic trials without purification.

In-solution reactions of JUK-20(Zn) with dienophiles:

200 mg of JUK-20(Zn) was placed in a glass vial containing 5 mL of N,N-dimethylformamide. 1.5 mmol of a dienophile was added and the mixture was left unstirred. Color change of the solid from red to yellow accompanied by nitrogen gas evolution was observed. The resulting products were filtered, washed with N,N-dimethylformamide and dried in air. By changing the vessel to a larger one, these syntheses were scaled up by a factor of 20. CHN analysis was conducted for desolvated samples.

{$[Zn_2(coh)_2(dpt\text{-}ala)_2]$·3$H_2O$}$_n$ (JUK-20(Zn)-ala), anal. calculated for $C_{64}H_{54}Zn_2N_{16}O_{15}$: C 52.04%; H 3.70%; N 15.17%. Found: C 52.36%, H 3.84%, N 15.18%.

{$[Zn_2(coh)_2(dpt\text{-}nol)_2]$·3$H_2O$}$_n$ (JUK-20(Zn)-nol), anal. calculated for $C_{72}H_{62}Zn_2N_{16}O_{15}$: C 55.06%; H 4.18%; N 14.07%. Found: C 54.94%, H 4.46%, N 14.10%.

{$[Zn_2(coh)_2(dpt\text{-}noh)_2]$·5.5$H_2O$}$_n$ (JUK-20(Zn)-noh), anal. calculated for $C_{74}H_{71}Zn_2N_{16}O_{17.5}$: C 55.72%; H 4.49%; N 14.05%. Found: C 55.85%, H 4.49%, N 14.48%.

The same procedure was repeated at a smaller scale using single crystals of JUK-20(Zn).

Determination of the JUK-20(Zn)-dienophile crystal structures was not possible due to single crystals disintegration. Therefore, by applying isomorphous Zn-Cd substitution, analogous reactions were carried out on JUK-20(Cd) single crystals, isostructurality was confirmed (see Fig. S4) and crystal structures were determined. PXRD, FT-IR and TGA measurements were performed (Fig. S2). About 40 mg of each pre-activated samples of the modified MOFs were dissolved in 0.8 mL of DMSO-d_6 with 5 drops of conc. D_2SO_4; the solutions were analyzed by ^1H NMR spectroscopy (Fig. S3).

Importantly, alternative *de novo* synthetic approaches to prepare JUK-20(Zn)-dienophile were also explored. The dpt-ala, dpt-nol and dpt-noh were utilized and a series of experiments at various temperatures, with different solvents and concentrations were carried out. Despite our best efforts, no crystal phases were obtained at all, including of JUK-20(Zn)-dienophile MOFs.

Mechanochemical iEDDA modifications of JUK-20(Zn):

80 mg of air-dried JUK-20(Zn) was placed together with 743 mg (1.50 mmol) of ala, 44 mg (0.40 mmol) of nol or 50 mg (0.40 mol) of noh dienophile in a 25 mL stainless steel grinding jar with 5 stainless steel balls (d = 10 mm). The mixtures were ground in a mixing mill with 25 Hz frequency for 50 minutes (for ala) or 20 minutes (for nol and noh). Yellowish solids after reaction were then washed with methanol and identified as phase-pure JUK-20(Zn)-dienophile MOFs (see Fig. S2). By changing the volume of the grinding jar, these syntheses were scaled up by a factor of 20.

Preparation of activated MOF samples:

Each MOF sample was immersed in analytical-grade methanol, which was replaced daily with a fresh portion for four days. After filtering, the sample was pre-activated by heating at 100 °C under 10 mbar for 1 h.

Details of mechanosyntheses and physical measurements

Unless otherwise noted, mechanochemical syntheses were performed on a MM200 Retsch mixing mill, using a 10 mL agate grinding jar and four 10mm diameter agate balls. Carbon, hydrogen, and nitrogen were determined by conventional microanalysis with the use of an Elementar Vario MICRO Cube elemental analyzer. FT-IR spectra were recorded on a Thermo Scientific Nicolet iS10 FT-IR spectrophotometer equipped with an iD7 diamond ATR attachment. Thermogravimetric analyzes (TGA) were performed at atmospheric pressure under flowing argon on a Mettler-Toledo TGA/SDTA 851^e instrument at a heating rate of 5°C min^{-1} in the temperature range of 25−400°C (sample weights were ca. 50 mg). ^1H and ^{13}C Nuclear Magnetic Resonance spectra were recorded on a Bruker Avance III 600 MHz spectrometer at 300 K; chemical shifts (δ, ppm) were determined in reference to internal residual signal from DMSO (2.49 ppm) or $CHCl_3$ (7.26 ppm). Single-crystal X-Ray Diffraction data were collected at 100 K using the Rigaku Oxford Diffraction Synergy-S four circle diffractometer, equipped with the Cu (1.54184 Å) Kα radiation source. PXRD patterns were recorded at room temperature (295 K) on a Rigaku Miniflex 600 diffractometer with Cu−Kα radiation (λ = 1.5418 Å) in a 2θ range from 3° to 45° with a 0.02° step at a scan speed of 3° min^{-1}. Variable-temperature powder X-ray diffraction (VT-PXRD) experiments were performed using Anton Paar BTS 500 heating stage from 30 to 300°C with a 10°C step. At each temperature, the samples were conditioned for 15 min before measurement. UV-vis reflectance spectra were recorded on a Shimadzu UV-2101PC spectrometer equipped with an ISR-260 attachment for solid samples. Sorption isotherms were measured on a Quantachrome Autosorb iQ-C-XR-XR EPDM instrument. Before measurements, the samples were washed several times with methanol (between each wash the sample was incubated in the solvent for 24 h) and then degassed at 100°C for 8 h. The sorption measurements were carried out at 77 K for N_2; at 273, 283 and 293 K for CO_2; at 293 K for D_2O and H_2O; and at 300 K for methanol, ethanol, and isopropanol vapors. Temperature control was afforded by using a liquid N_2 bath (77 K) or chilled water (273-300 K). Solid-state photoluminescent characterization for all reported compounds was performed using an FS5 spectrofluorometer (Edinburgh Instruments) equipped with a Xe arc lamp (150 W, excitation spectra) serving as excitation source, and a Hamamatsu photomultiplier of the R928P type as a detector. The Fluoracle softwere and OriginPro 2021b program were employed for the background corrections and a smoothing procedure. Humidity-dependent measurements were performed in situ using a home-made setup employing HG-100 RH humidity generator (L&C Science and Technology).

Calculations of green metrics factors

E-factor calculations

$$E_{coh,ISS} = \frac{m_{carbohydrazide}+m_{aldehyde}+m_{H2O}+m_{EtOH}-m_{coh}}{m_{coh}} = \frac{(1.20+0.360+30.0+39.5-1.15)g}{1.15\ g} \approx \mathbf{60.7}$$

$$E_{coh,MS} = \frac{m_{carbohydrazide}+m_{aldehyde}+m_{EtOH}-m_{coh}}{m_{coh}} = \frac{(60.0+18.0+30.0+78.9-70.8)mg}{70.8\ mg} \approx \mathbf{1.64}$$

$$E_{JUK-20(Zn),ISS} = \frac{m_{Zinc\,salt}+m_{coh}+m_{dpt}+m_{DMF}+m_{MeOH}-m_{JUK-20(Zn)}}{m_{JUK-20(Zn)}} = \frac{(0.446+0.534+0.354+127+11.9-0.881)g}{0.881\ g} \approx \mathbf{159}$$

$$E_{JUK-20(Zn),MS} = \frac{m_{Zinc\,salt}+m_{coh}+m_{dpt}+m_{DMF}-m_{JUK-20(Zn)}}{m_{JUK-20(Zn)}} = \frac{(22.0+35.6+23.6+76.7-86.4)mg}{86.4\ mg} \approx \mathbf{0.828}$$

$$E_{JUK-20(Zn)-ala,ISS} = \frac{m_{JUK-20(Zn)}+m_{ala}+m_{DMF}-m_{JUK-20(Zn)-ala}}{m_{JUK-20(Zn)-ala}} = \frac{(0.200+0.0870+4.72-0.164)g}{0.164\ g} \approx \mathbf{29.5}$$

$$E_{JUK-20(Zn)-ala,MS} = \frac{m_{JUK-20(Zn)}+m_{ala}-m_{JUK-20(Zn)-ala}}{m_{JUK-20(Zn)-ala}} = \frac{(80.0+743-65.7)mg}{65.7\ mg} \approx \mathbf{11.5}$$

** ISS refers to in-solution synthesis, whereas MS refers to mechanosynthesis*

Process mass intensity (PMI) calculations

$$PMI_{coh,ISS} = \frac{m_{carbohydrazide}+m_{aldehyde}+m_{H2O}+m_{EtOH}}{m_{coh}} = \frac{(1.20+0.360+30.0+39.5)g}{1.15\ g} \approx \mathbf{61.7}$$

$$PMI_{coh,MS} = \frac{m_{carbohydrazide}+m_{aldehyde}+m_{EtOH}}{m_{coh}} = \frac{(60.0+18.0+30.0+78.9)mg}{70.8\ mg} \approx \mathbf{2.64}$$

$$PMI_{JUK-20(Zn),ISS} = \frac{m_{Zinc\,salt}+m_{coh}+m_{dpt}+m_{DMF}+m_{MeOH}}{m_{JUK-20(Zn)}} = \frac{(0.446+0.534+0.354+127+11.9)g}{0.881\ g} \approx \mathbf{160}$$

$$PMI_{JUK-20(Zn),MS} = \frac{m_{Zinc\,salt}+m_{coh}+m_{dpt}+m_{DMF}}{m_{JUK-20(Zn)}} = \frac{(22.0+35.6+23.6+76.7)mg}{86.4\ mg} \approx \mathbf{1.83}$$

$$PMI_{JUK-20(Zn)-ala,ISS} = \frac{m_{JUK-20(Zn)}+m_{ala}+m_{DMF}}{m_{JUK-20(Zn)-ala}} = \frac{(0.200+0.0870+4.72)g}{0.164\ g} \approx \mathbf{30.5}$$

$$PMI_{JUK-20(Zn)-ala,MS} = \frac{m_{JUK-20(Zn)}+m_{ala}}{m_{JUK-20(Zn)-ala}} = \frac{(80.0+743)mg}{65.7\ mg} \approx \mathbf{12.5}$$

Reaction mass efficiency (RME) calculations

$$RME_{coh,ISS} = \frac{m_{coh}}{m_{carbohydrazide}+m_{aldehyde}} = \frac{1.15\ g}{(1.20+0.360)g} \approx \mathbf{0.737}$$

$$RME_{coh,MS} = \frac{m_{coh}}{m_{carbohydrazide}+m_{aldehyde}} = \frac{70.8\ mg}{(60.0+18.0)mg} \approx \mathbf{0.907}$$

$$RME_{JUK-20(Zn),ISS} = \frac{m_{JUK-20(Zn)}}{m_{Zinc\,salt}+m_{coh}+m_{dpt}+m_{DMF}} = \frac{0.881\ g}{(0.446+0.534+0.354+127)g} \approx \mathbf{0.00684}$$

$$RME_{JUK-20(Zn),MS} = \frac{m_{JUK-20(Zn)}}{m_{Zinc\,salt}+m_{coh}+m_{dpt}+m_{DMF}} = \frac{86.4\ mg}{(22.0+35.6+23.6+76.7)mg} \approx \mathbf{0.547}$$

$$RME_{JUK-20(Zn)-ala,ISS} = \frac{m_{JUK-20(Zn)-ala}}{m_{JUK-20(Zn)}+m_{ala}} = \frac{0.164\ g}{(0.200+0.0870)g} \approx \mathbf{0.571}$$

$$RME_{JUK-20(Zn)-ala,MS} = \frac{m_{JUK-20(Zn)-ala}}{m_{JUK-20(Zn)}+m_{ala}} = \frac{65.7\ mg}{(80.0+743)mg} \approx \mathbf{0.0798}$$

Energy efficiency calculations

$$E_{coh,ISS} = \frac{\frac{1}{3}h\cdot 1020W}{1.15g} = \frac{0.340kWh}{1.15g} \approx \mathbf{296}\,\frac{\mathbf{kWh}}{\mathbf{kg}} \approx \mathbf{1\,070}\,\frac{\mathbf{MJ}}{\mathbf{kg}}$$

$$E_{coh,MS} = \frac{\frac{1}{3}h\cdot 100W}{2\cdot 0.0708g} = \frac{0.0333kWh}{0.142g} \approx \mathbf{235}\,\frac{\mathbf{kWh}}{\mathbf{kg}} \approx \mathbf{845}\,\frac{\mathbf{MJ}}{\mathbf{kg}}$$

$$\%Energy\ saved = \frac{E_{coh,ISS}-E_{coh,MS}}{E_{coh,SS}}\cdot 100\% \approx \mathbf{20.7\%}$$

$$E_{JUK-20(Zn),ISS} = \frac{48h\cdot 2000W}{0.881g} = \frac{96.0kWh}{0.881g} \approx \mathbf{109\,000}\,\frac{\mathbf{kWh}}{\mathbf{kg}} \approx \mathbf{392\,000}\,\frac{\mathbf{MJ}}{\mathbf{kg}}$$

$$E_{JUK-20(Zn),MS} = \frac{\frac{1}{2}h\cdot 100W}{0.0864\ g} = \frac{0.0500kWh}{0.0864\ g} \approx \mathbf{579}\,\frac{\mathbf{kWh}}{\mathbf{kg}} \approx \mathbf{2\,080}\,\frac{\mathbf{MJ}}{\mathbf{kg}}$$

$$\%Energy\ saved = \frac{E_{JUK-20(Zn),ISS}-E_{JUK-20(Zn),MS}}{E_{JUK-20(Zn),SS}}\cdot 100\% \approx \mathbf{99.5\%}$$

$$E_{JUK-20(Zn)-ala,ISS} = \frac{2h\cdot 1020W}{0.164\ g} = \frac{2.04kWh}{0.164\ g} \approx \mathbf{12\,400}\,\frac{\mathbf{kWh}}{\mathbf{kg}} \approx \mathbf{44\,800}\,\frac{\mathbf{MJ}}{\mathbf{kg}}$$

$$E_{JUK-20(Zn)-ala,MS} = \frac{\frac{5}{6}h\cdot 100W}{0.0657\ g} = \frac{0.0833kWh}{0.0657\ g} \approx \mathbf{1\,270}\,\frac{\mathbf{kWh}}{\mathbf{kg}} \approx \mathbf{4\,570}\,\frac{\mathbf{MJ}}{\mathbf{kg}}$$

$$\%Energy\ saved = \frac{E_{JUK-20(Zn)-ala,ISS}-E_{JUK-20(Zn)-ala,MS}}{E_{JUK-20(Zn),SS}}\cdot 100\% \approx \mathbf{89.8\%}$$

Figures and Tables

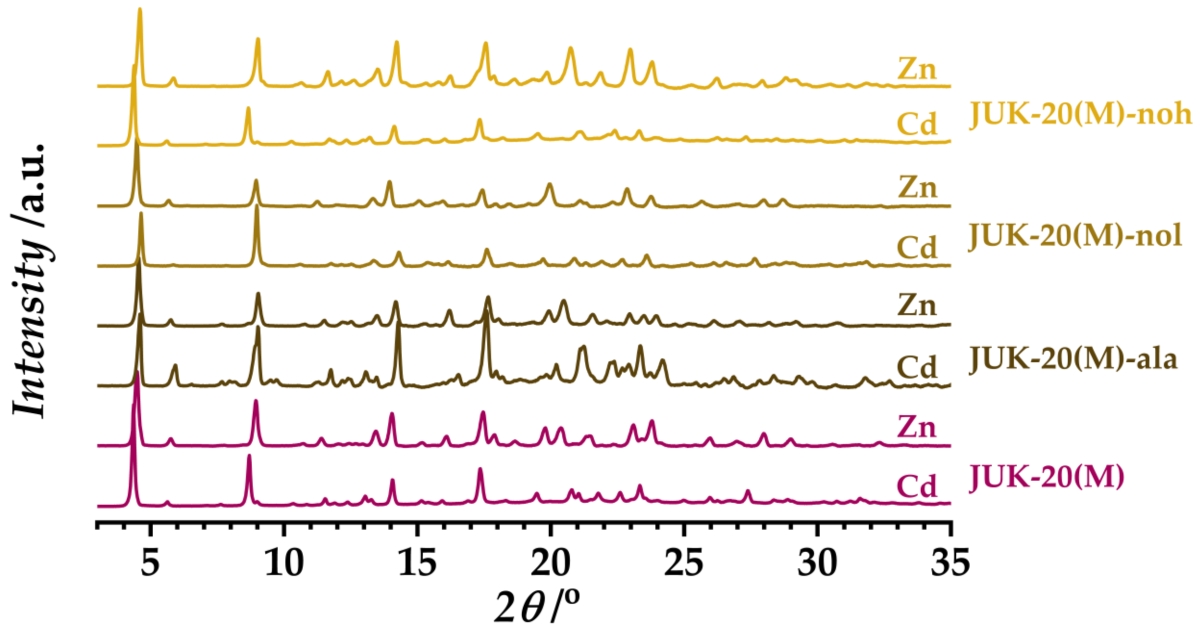

Figure S1. Comparison of powder XRD patterns measured for JUK-20(Zn)-x materials and their cadmium analogues as evidence of their isostructurality.

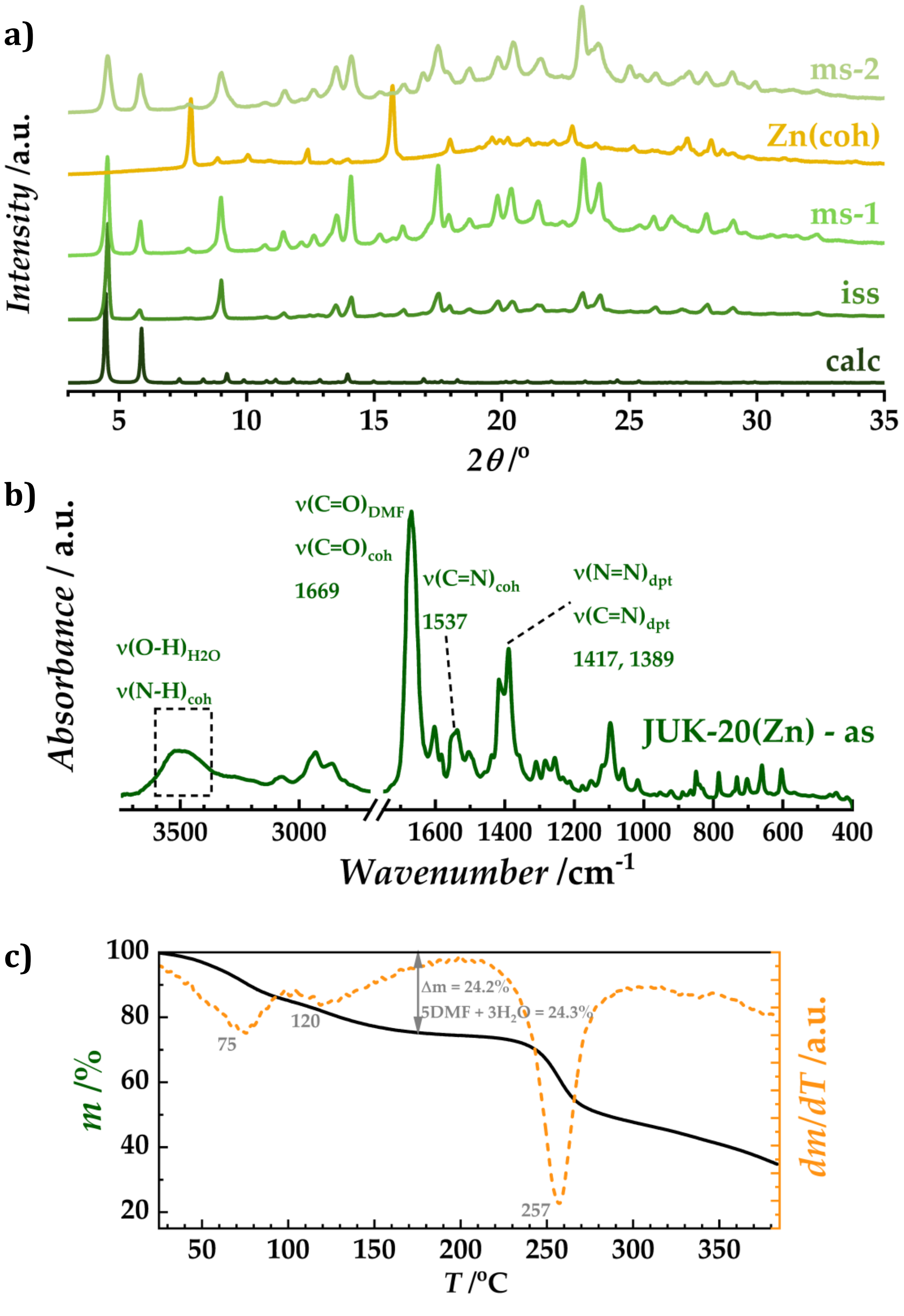

Figure S2. Characterization of JUK-20(Zn): powder X-ray diffraction patterns (a) for samples obtained from in-solution synthesis (*iss*) and mechanosynthesis (in one-step variant, *ms-1* or three-step variant, *ms-2*), compared to pattern calculated from SC-XRD data (*calc*) and pattern measured for Zn(coh) in the second stage of mechanosynthesis. FT-IR spectrum (b) and TG curve (c) for iss sample.

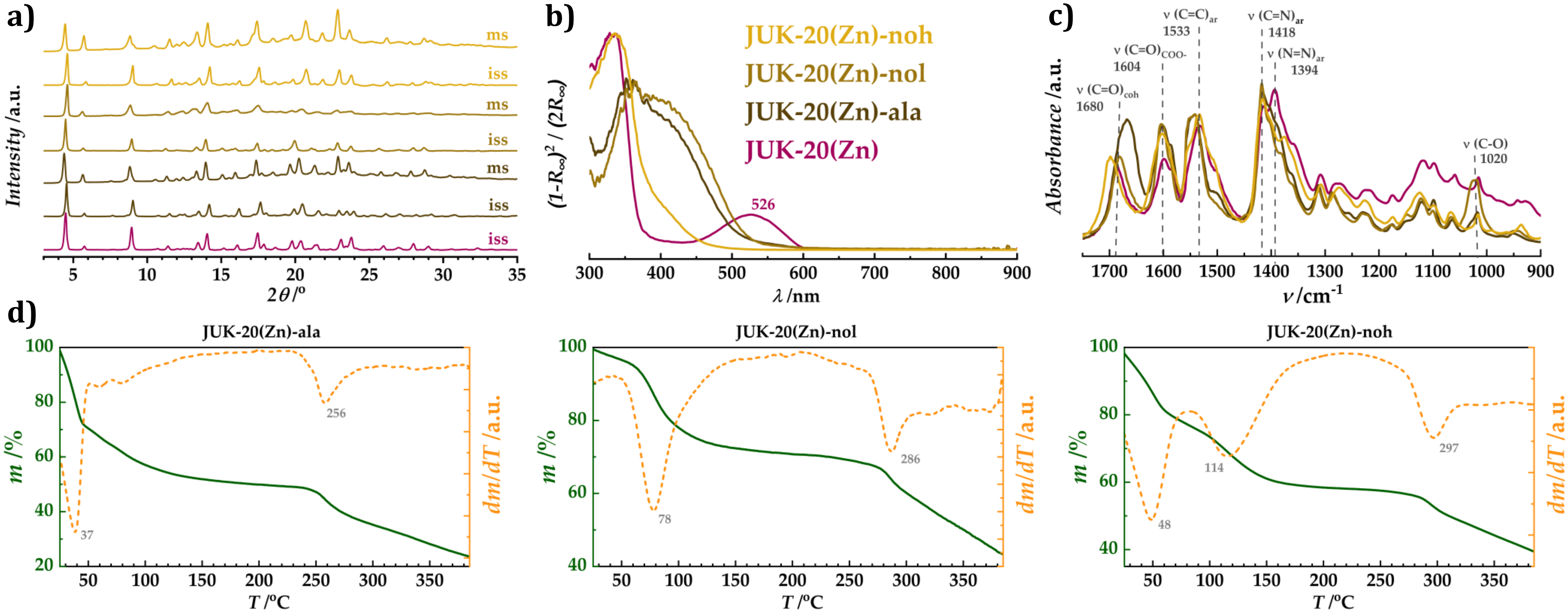

Figure S3. Characterization of the JUK-20(Zn)-dienophile series: powder X-ray patterns (a) for samples obtained in-solution (*iss*) or mechanochemically (*ms*). UV-vis reflectance spectra (b) and FT-IR spectra (c) for in-solution obtained samples. Characterization of the JUK-20(Zn)-dienophile series by TG (for methanol-exchanged samples).

Comments to Fig. S2: PXRD patterns (a) confirm the retention of crystallinity and very slight changes in crystal cell parameters. UV-vis reflectance spectra (b) reveal a complete disappearance of the band at 526 nm upon CPSM. FT-IR spectra (c) were measured for activated samples and normalized by the 1533 cm^{-1} band (aromatic C=C stretch). The absorption band connected with N=N aromatic bond stretches (1394 cm^{-1}) is weakened after CPSM. A significant change in 1020 cm^{-1} region, related to the appearance of C-O bond, is also noticeable. TG curves (d) show a stepwise mass loss with a first plateau upon removal of guests, prior to thermal degradation of the frameworks. A slight gradual increase of thermal stability (by ca. 30 and 40°C) of JUK-20(Zn)-nol and -noh networks can be observed, as compared to parent JUK-20(Zn) and JUK-20(Zn)-ala materials.

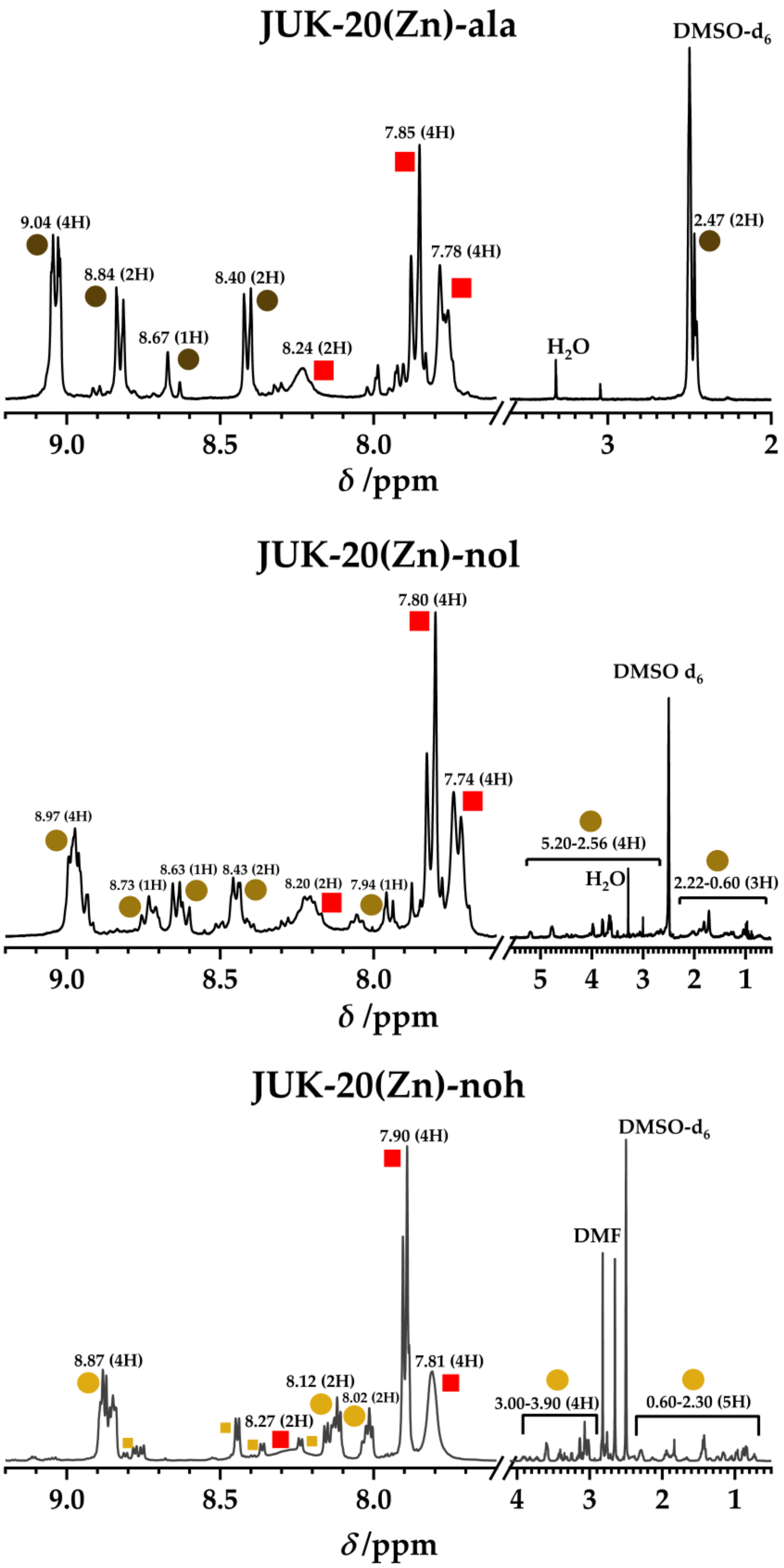

Figure S4. ^{1}H NMR spectra for JUK-20(Zn)-dienophile networks digested in deuterated sulfuric acid/dimethylsulfoxide-d_6.

Comments to Fig. S3: The peaks of the dpt-dienophile ligands are labeled with brown circles. Peaks of coh ligands are labeled with red squares. Residual non-deuterated DMSO and non-removed DMF peaks are labelled. For JUK-20(Zn)-noh, minor contribution for an alternative product (1,4-dihydrodiazine instead of 4,5-dihydrodiazine) is labeled with brown squares.[1]

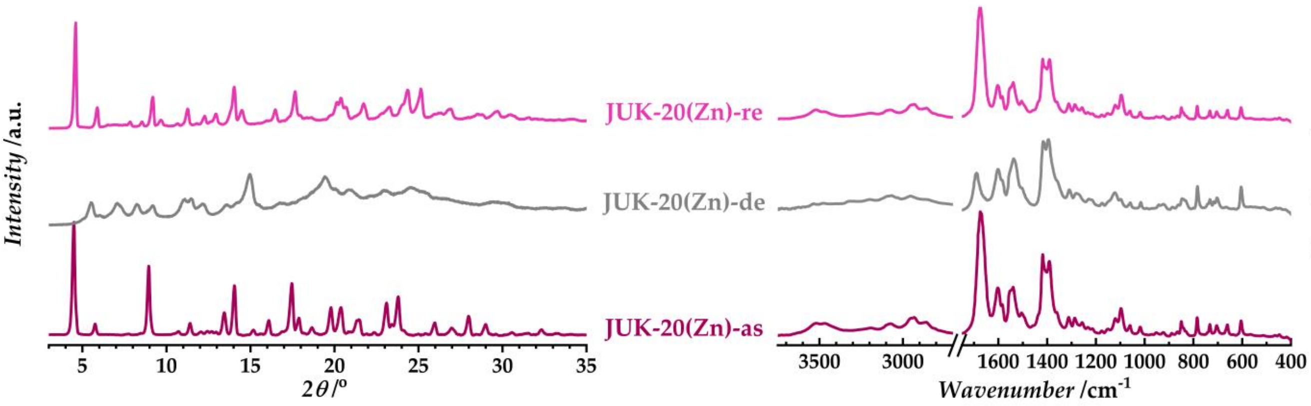

Figure S5. Desolvation-resolvation of solid JUK-20(Zn): powder X-ray patterns, including resolvation in DMF (left), FT-IR spectra, including resolvation in DMF (right). *As*: as-synthesized, *de*: desolvated, and *re*: resolvated.

Table S1. Structural analysis of **JUK-20(M)-x** series models determined by Zeo++ simulations and nitrogen adsorption measurements.

Material	Unit cell Volume (Å³)	Density (g/cm³)	S_{BET} (m²/g)	Specific geometric surface area (m²/g)	Specific pore volume simulated (cm³/g)	Void fraction
**JUK-20(Cd)**[a]	5154.90	0.903	n/a	1529	0.533	0.481
**JUK-20(Zn)**	4899.24	0.886	5[b]	1584	0.500	0.443
**JUK-20(Cd)-ala**	5155.29	0.939	n/a	1317	0.439	0.412
**JUK-20(Zn)-ala**	*4899.61*	*0.924*	28[b]	n/a	*0.410*	*0.379*
**JUK-20(Cd)-nol**	5270.11	0.986	n/a	1213	0.357	0.352
**JUK-20(Zn)-nol**	*5008.74*	*0.976*	87[c]	n/a	*0.332*	*0.324*
**JUK-20(Cd)-noh**[a]	5332.74	1.008	1026	1289	0.400	0.393
**JUK-20(Zn)-noh**	*5068.25*	*0.999*	1051[d]	n/a	*0.362*	*0.362*

[a] Data from the previous report.[1]
[b] BET specific surface area calculated in the p/p_0 pressure range of 0.05 - 0.25.
[c] BET specific surface area calculated in the p/p_0 pressure range of 0.10 - 0.40
[d] BET specific surface area calculated in the p/p_0 pressure range of 0.001 - 0.01

Comments to **Table S1**: Unit cell volumes and void fractions for experimental crystallographic data (normal font) and estimated (italic font). The transition from JUK-20(Cd) to (Zn) resulted in a decrease of unit cell volume and a decrease by 8.58% in void fraction. The same decreases were used to estimate unit cell volume and void fractions for the remaining zinc MOFs.
The Brunauer-Emmett-Teller (BET) theory was used to calculate the specific surface areas of the materials obtained. For all isotherm analyses, we ensured that the two consistency criteria described by Rouquerol et al.[4] and Walton et al.[5] were satisfied.
The Zeo++ software[6] was used for the structural analysis of prepared MOF models. The specific surface area and pore volume of prepared MOF models were calculated using a spherical probe with a radius of 1.86 Å.[7]

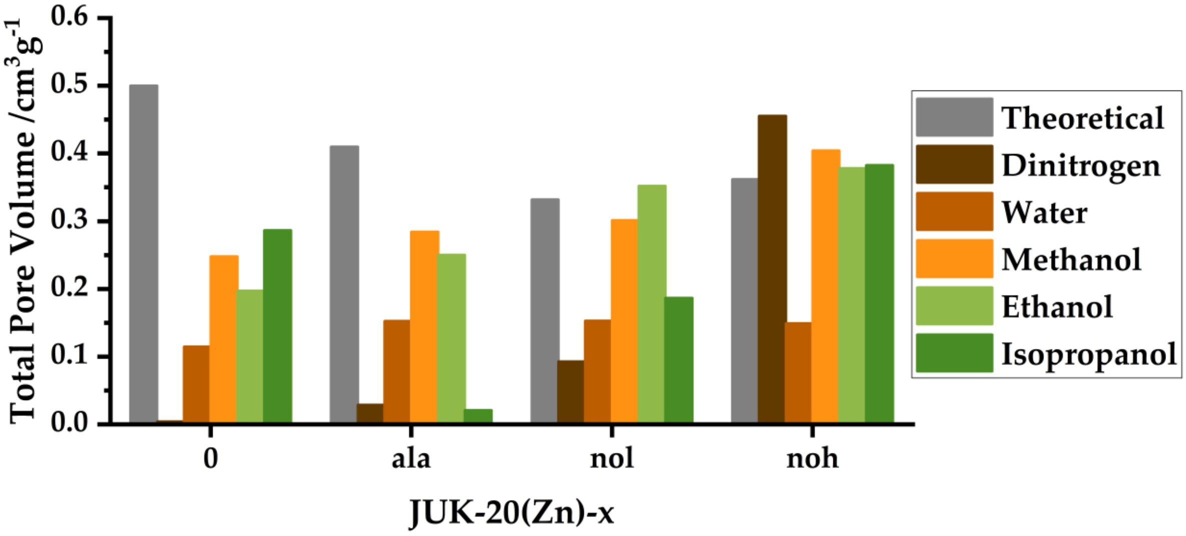

Figure S6. Void volume occupied by different adsorbates in the JUK-20(Zn)-x series

Comments to **Figure S6**: The total pore volume was calculated from single-point adsorbate uptake at p/p_0 = 0.95 for dinitrogen and at p/p_0 = 0.90 for the remaining adsorbates. The Gurvich rule was utilized for estimation of the volume occupied by adsorbate. Theoretical total pore volumes were calculated with Zeo++ (see Table S1).

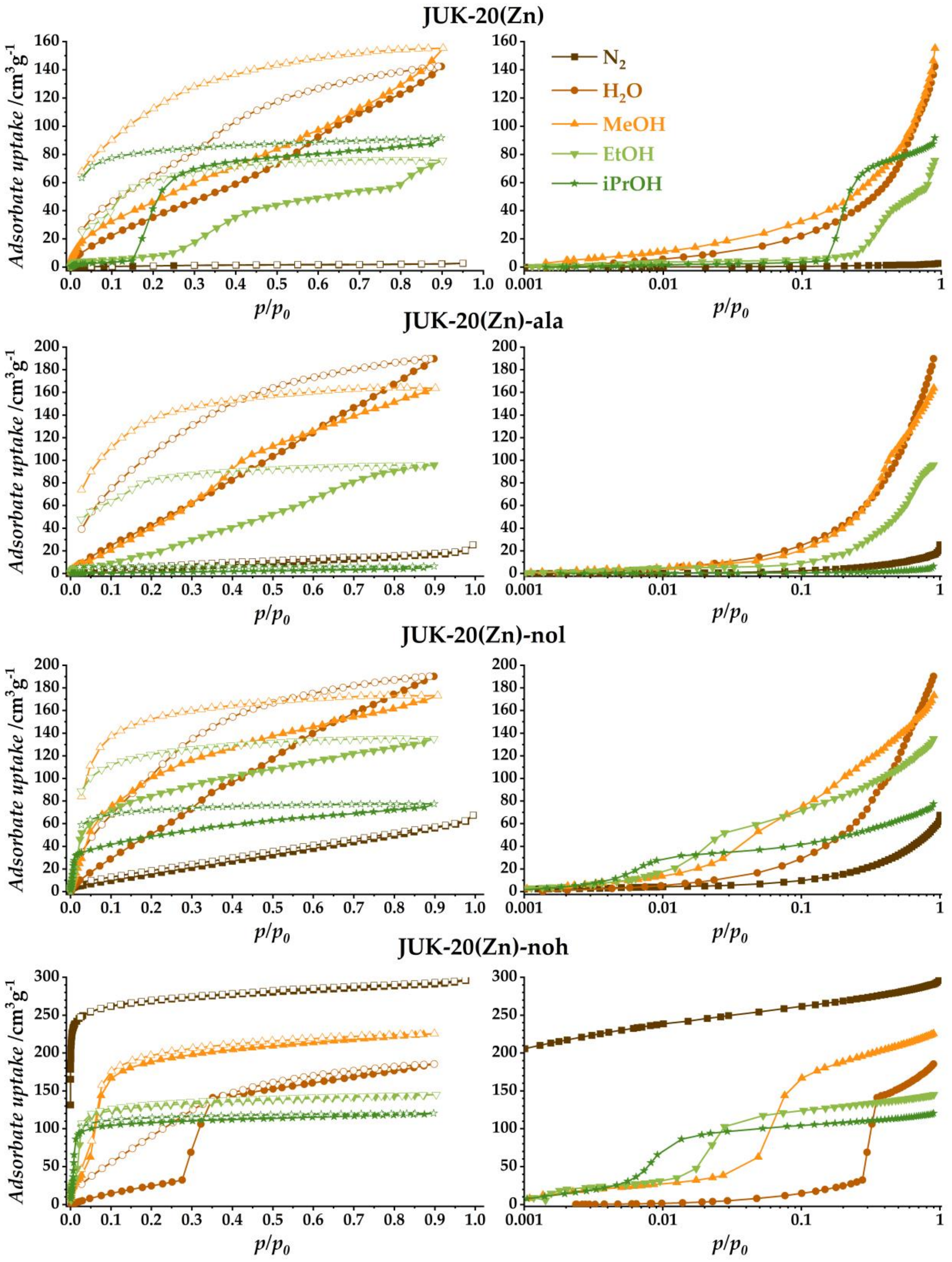

Figure S7. Adsorption (closed symbols) and desorption (open symbols) isotherms for materials studied, measured for different adsorbates. Linear scale (left) and logarithmic scale (right) presented. For logarithmic scale, desorption isotherms are removed for clarity.

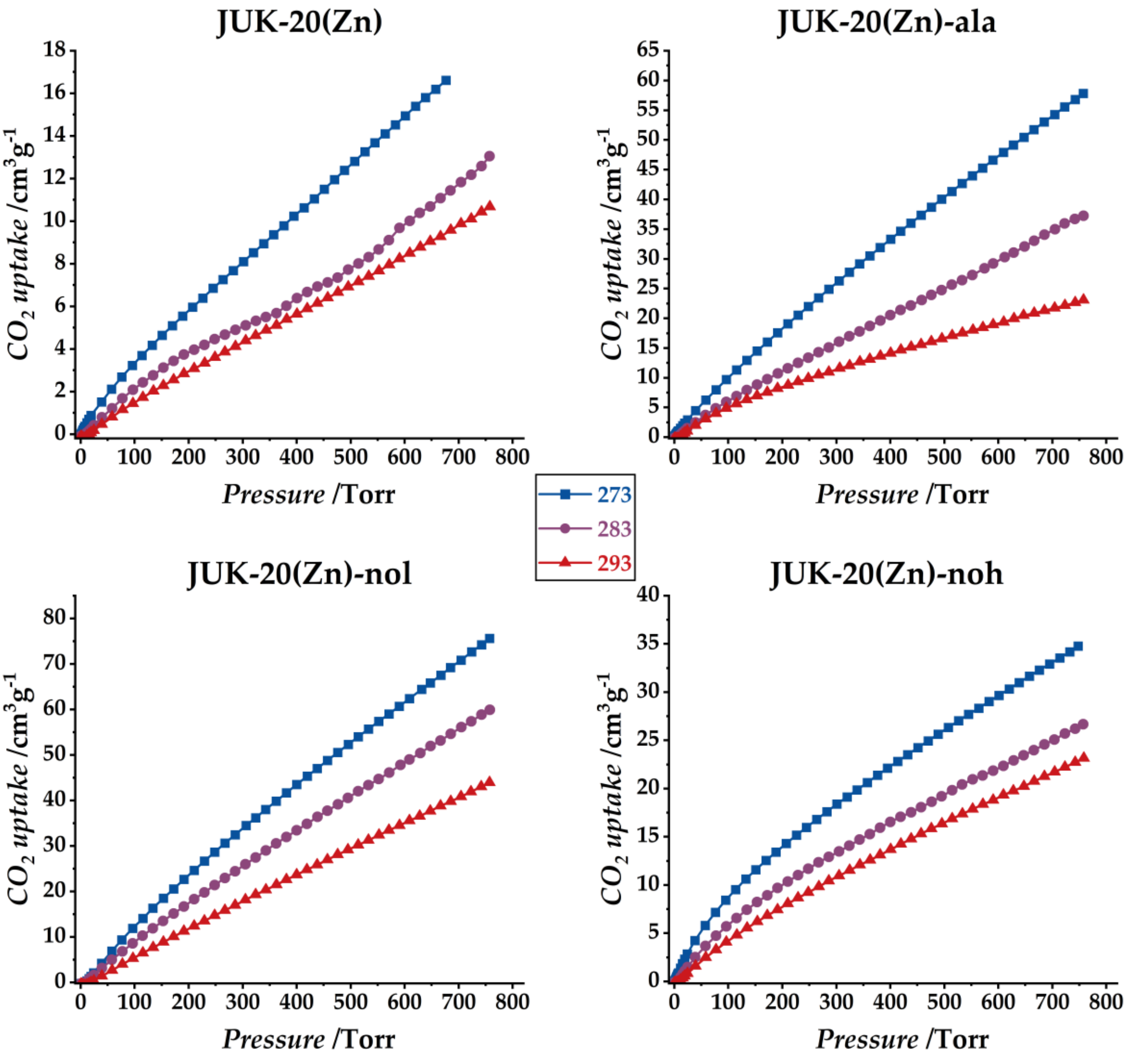

Figure S8. CO_2 heat of adsorption studies. Carbon dioxide adsorption isotherms at 273, 283 and 293 K for JUK-20(Zn)-x materials are presented. The Freundlich model was utilized to calculate the isosteric heat of adsorption.

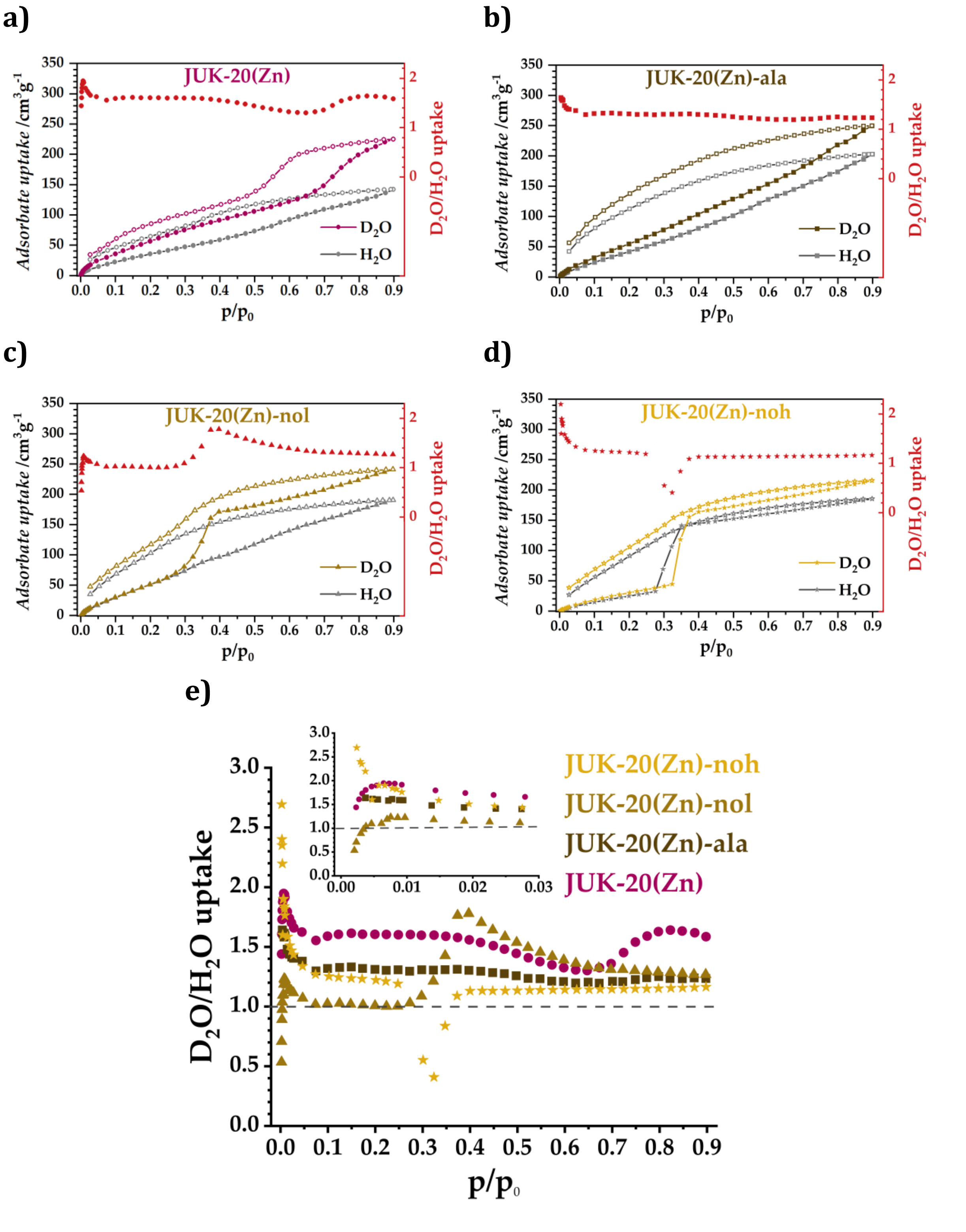

Figure S9. D_2O adsorption isotherms (293 K) compared with H_2O adsorption isotherms (293 K) for all MOFs (a-d). Estimation of materials selectivity for deuterated water adsorption over H_2O (e).

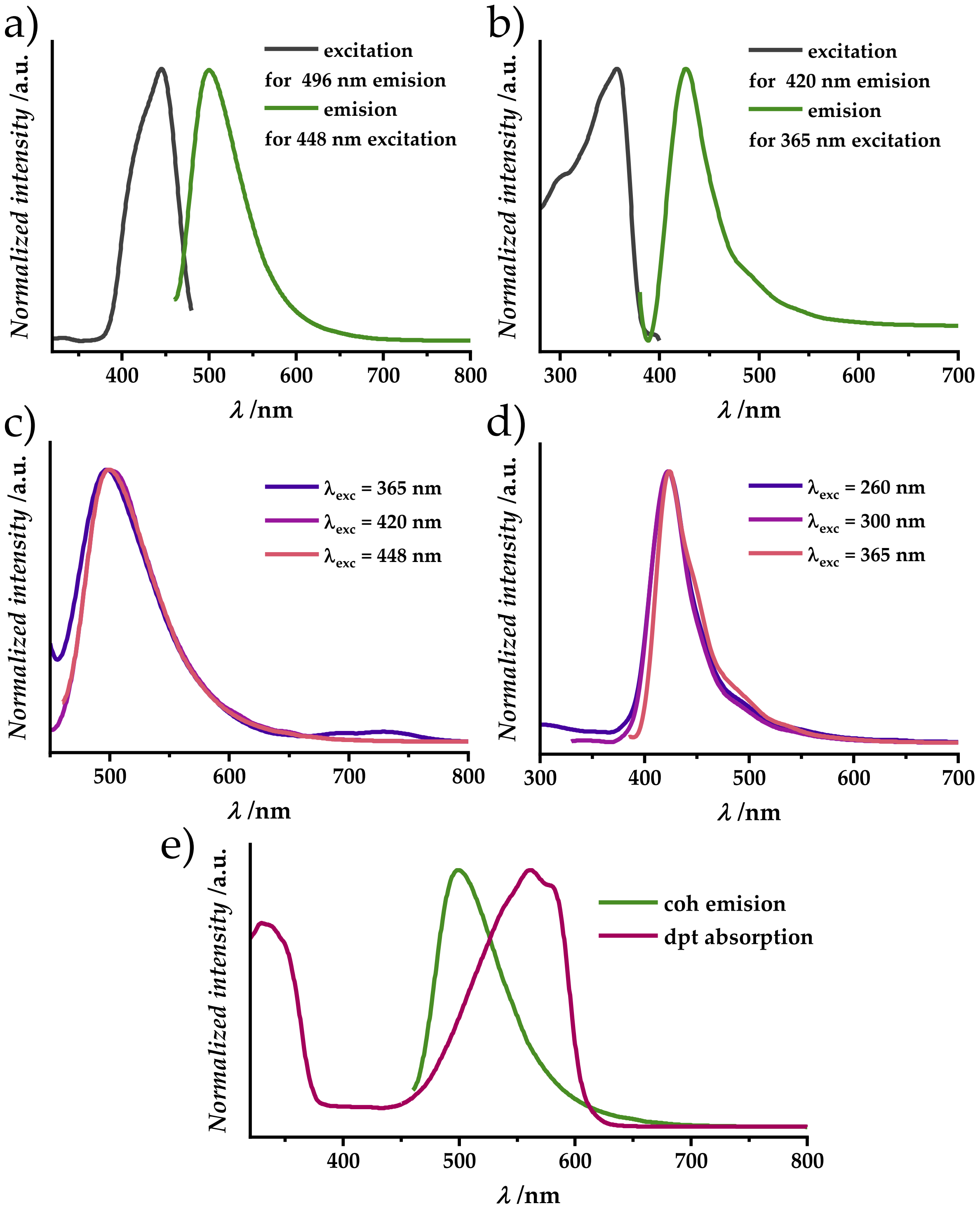

Figure S10. Emission (green) and excitation (black) spectra for the coh ligand measured at 293 K (a) and 77 K (b). Normalized emission spectra for the coh ligand measured 293 K (c) and 77 K (d) at different excitation wavelengths. Comparison of the coh ligand emission spectrum at 293 K for 448 nm excitation wavelength (green) and reflectance spectrum of the dpt ligand (violet) (e). All spectra were collected for solid-state samples.

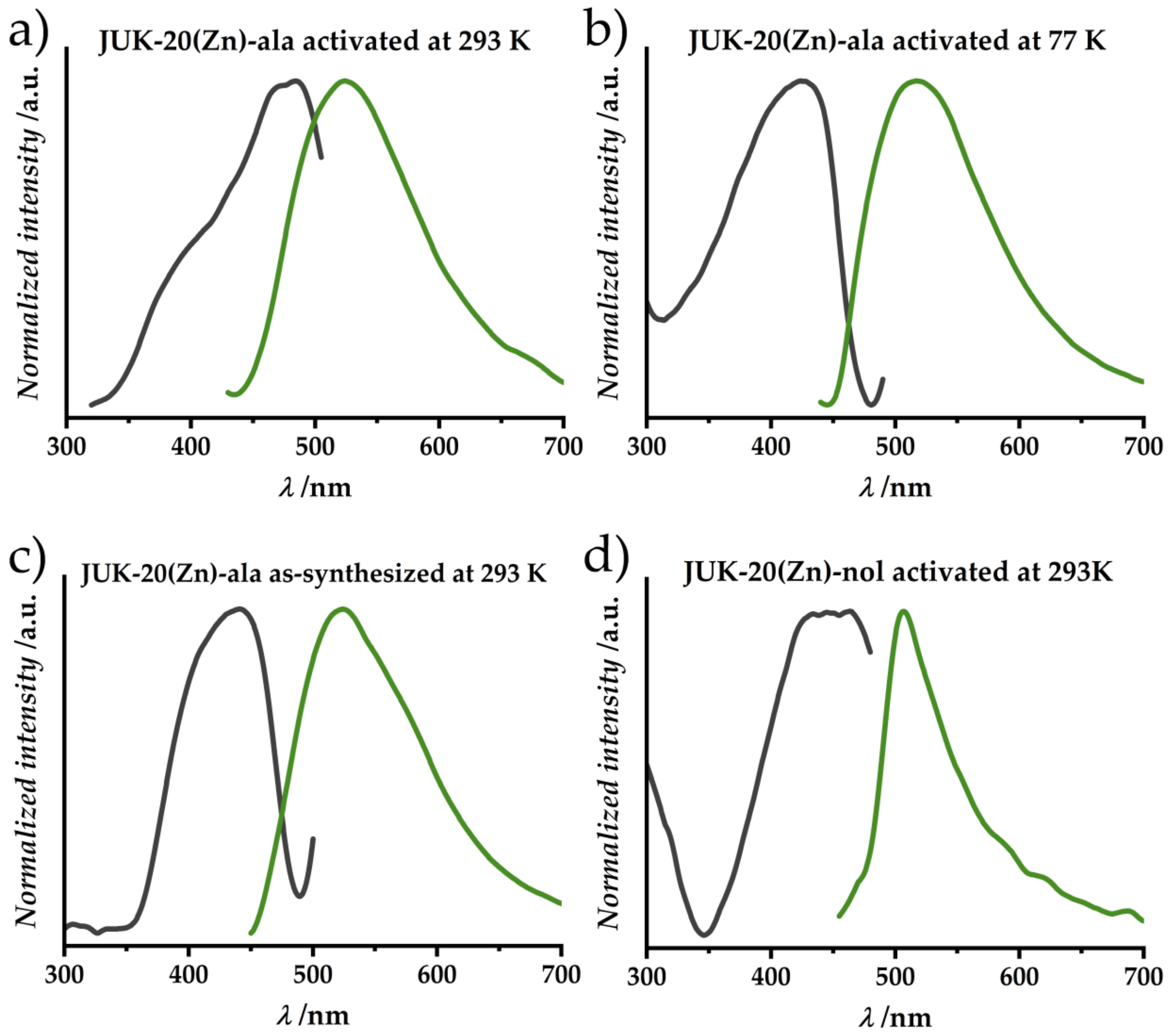

Figure S11. Excitation (black) and emission (green) spectra for activated JUK-20(Zn)-ala material (at 293 K – a, at 77 K – b), for as-synthesized JUK-20(Zn)-ala measured at 293 K (c) and for activated JUK-20(Zn)-nol at 293 K.
(a) – excitation spectra for λ_{em} = 525 nm, emission spectra for λ_{exc} = 400 nm;
(b) – excitation spectra for λ_{em} = 510 nm, emission spectra for λ_{exc} = 420 nm;
(c) – excitation spectra for λ_{em} = 520 nm, emission spectra for λ_{exc} = 440 nm;
(d) – excitation spectra for λ_{em} = 505 nm, emission spectra for λ_{exc} = 440 nm.

Table S2. Comparison of luminescence parameters for materials studied. All data were collected at 293 K (in case of no emission, additional measurement at 77 K confirmed too weak luminescence to be measured). For luminescent MOFs, their specific phases (as - as-synthesized, act - activated) are indicated.

	dpt	dpt-ala/ dpt-nol/ dpt-noh	coh	Zn(coh)	JUK-20(Zn)	JUK-20(Zn)-eve	JUK-20(Zn)-ala		JUK-20(Zn)-nol		JUK-20(Zn)-noh
							as	*act*	*as*	*act*	
λ^{exc}_{max} (nm)	---	---	446 (496 nm em)	420 (468 nm em)	---	---	442 (524 nm em)	485 (525 nm em)	---	443 (507 nm em)	---
λ^{em}_{max} (nm)	---	---	500 (448 nm exc)	468 (420 nm exc)	---	---	524 (442 nm exc)	525 (485 nm exc)	---	507 (443 nm exc)	---

Comments to Table S2: no value means no emission at measurement conditions were detected. Comparison of dpt, dpt-dienophile, coh, JUK-20(Zn) and JUK-20(Zn)-ala confirms the origin of JUK-20(Zn)-ala luminescence (coh ligand). Comparison between JUK-20(Zn)-eve, -ala, -nol and -noh confirms two requirements for coh ligand luminescence: MOF flexibility (JUK-20(Zn)-ala vs -noh) and the presence of OH group (JUK-20(Zn)-ala vs -eve).

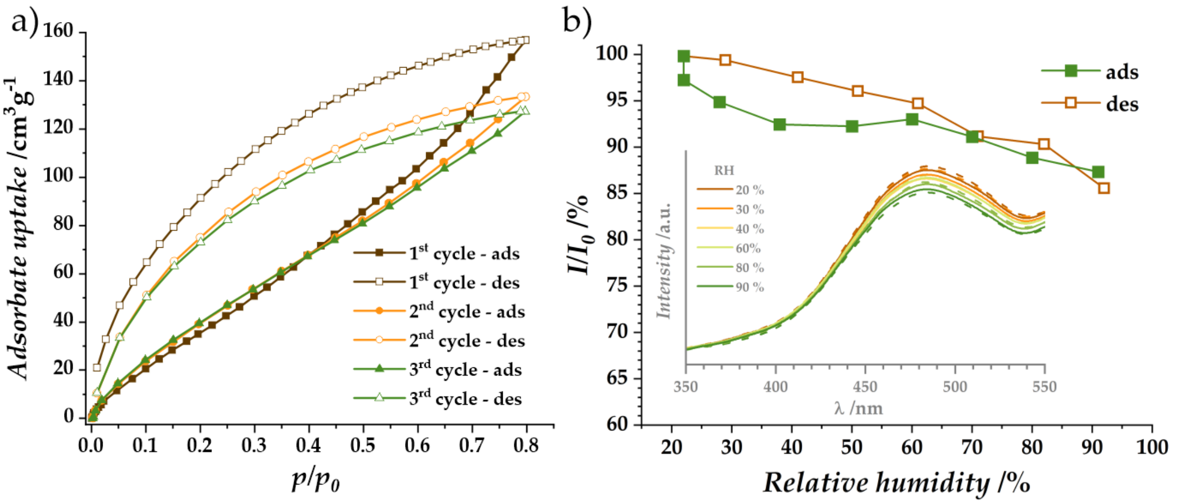

Figure S12. Consecutive cycles of JUK-20(Zn)-ala water vapor sorption (a). Excitation isotherm for JUK-20(Zn)-ala in varying humidity (a). Emission spectra for selected points of adsorption (straight line) and desorption (dashed line) (b).

Table S3. Optical images (under sunlight and UV light) of solid products obtained during total mechanosynthesis of JUK-20(Zn)-ala.

Material	Sunlight	UV-light
**4-formylbenzoic acid + carbohydrazone**	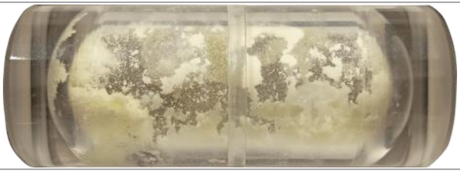	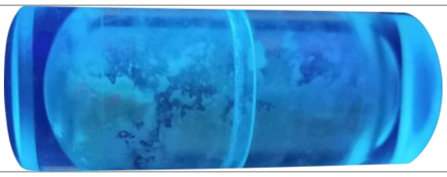
$\downarrow \boldsymbol{grinding}$		
**Coh ligand**	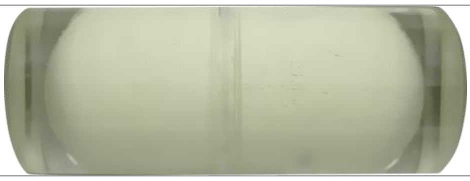	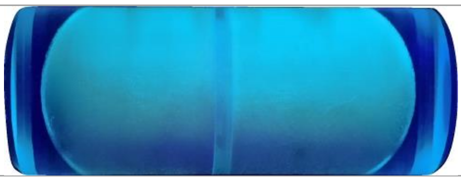
$\downarrow + \boldsymbol{Zn(OAc)_2 \cdot 2H_2O, grinding}$		
**Zn(coh)**	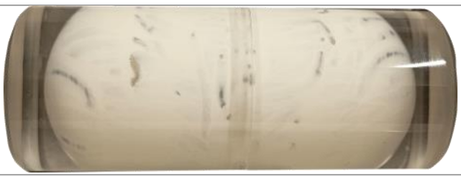	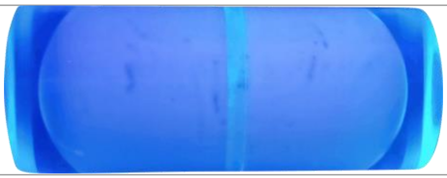
$\downarrow + \boldsymbol{dpt\ ligand, grinding}$		
**JUK-20(Zn)**	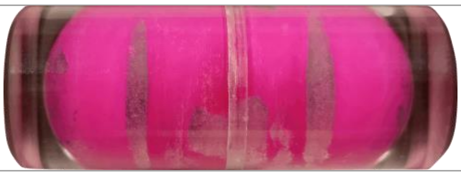	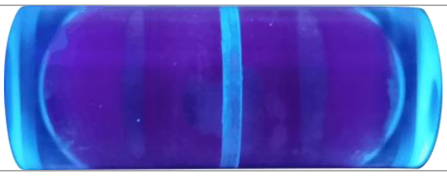
$\downarrow + \boldsymbol{ala\ dienophile, grinding}$		
**JUK-20(Zn)-ala**	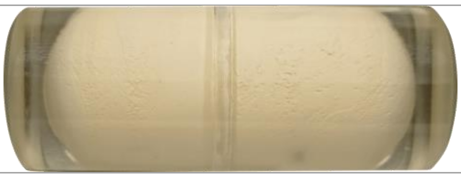	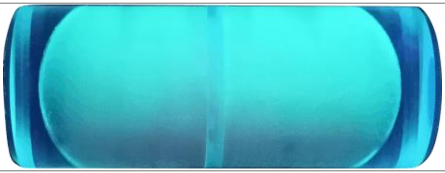

a)

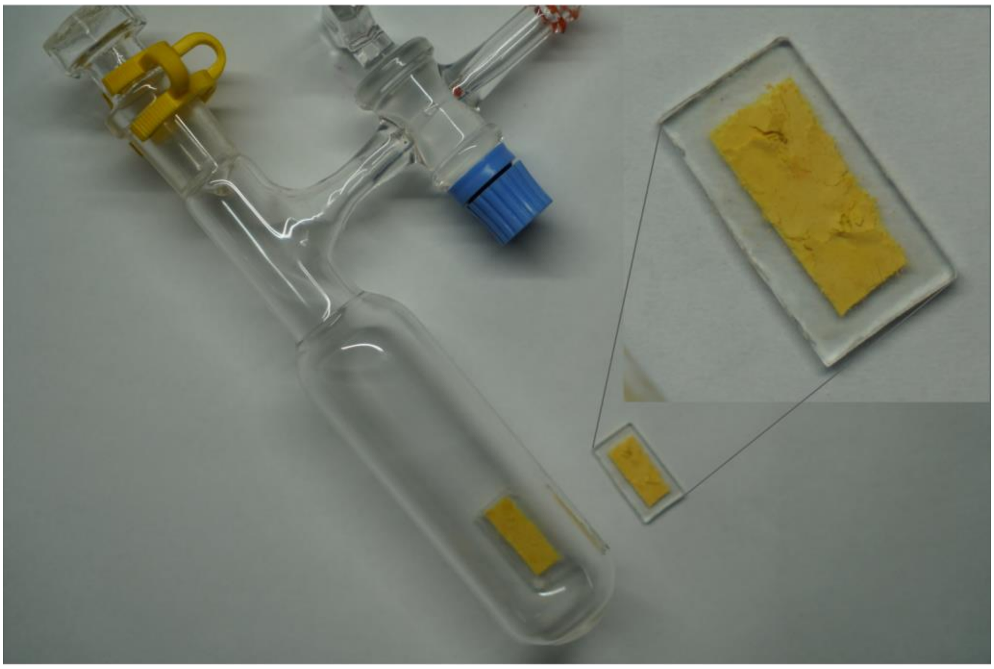

b)

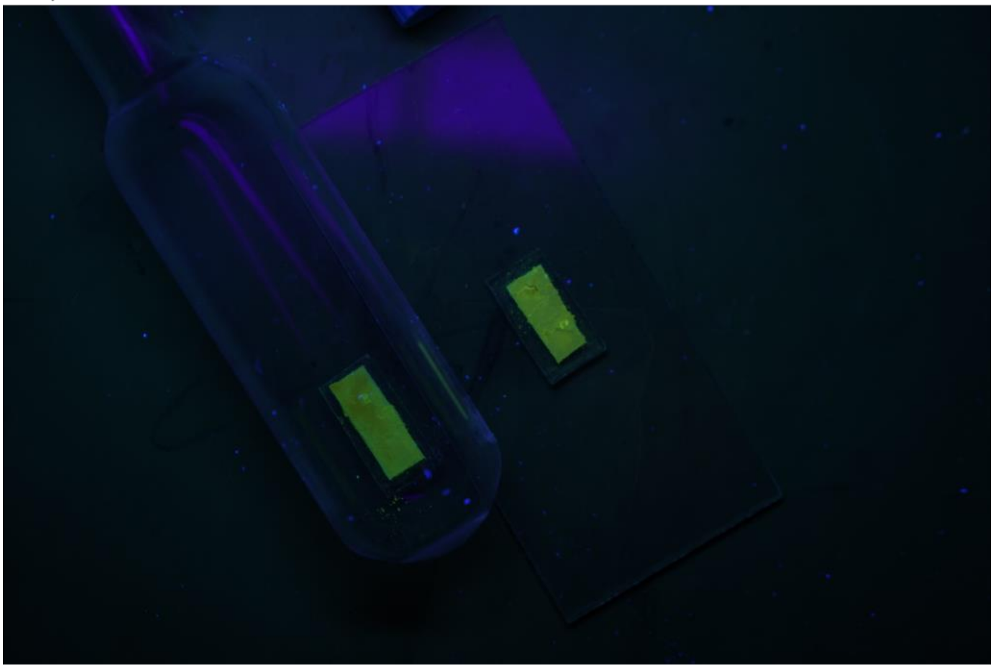

Figure S13. The photograph of a planar sensor including both activated (Schlenk tube, left side) and humidified (at RH=94%, right side) polycrystalline samples of JUK-20(Zn)-ala under sunlight (a) and UV light (b) positioned on a glass support.

Structural analysis details

Identification code	JUK-20(Zn)	JUK-20(Cd)-ala	JUK-20(Cd)-nol
Empirical formula	$C_{70}H_{70}N_{24}O_{17}Zn_2$	$C_{68.82}H_{61.63}Cd_2N_{18}O_{13.82}$	$C_{72}H_{58}Cd_2N_{16}O_{12}$
Formula weight	1650.24	1586.65	1564.14
Temperature	100(2) K	100(2) K	100(2) K
Wavelength	1.54184 Å	1.54184 Å	1.54184 Å
Crystal system	Triclinic	Triclinic	Triclinic
Space group	P -1	P -1	P -1
Unit cell dimensions	a = 19.9132(2) Å<br>b = 15.3560(2) Å<br>c = 17.41150(10)Å<br>α = 84.1850(10)°<br>β = 85.5650(10)°<br>γ = 67.7850(10)°	a = 20.4845(4) Å<br>b = 15.7952(3) Å<br>c = 16.9734(2) Å<br>a= 86.299(1)°<br>b= 85.162(1)°<br>g = 70.524(2)°	a = 15.8523(3) Å<br>b = 16.9648(2) Å<br>c = 20.5155(3) Å<br>α = 85.1010(10)°<br>β = 73.8120(10)°<br>γ = 85.4240(10)°
Volume (Å^3)	4899.24(9)	5155.29(16)	5270.11(14)
Z	2	2	2
Density (calculated)	1.119 Mg/m^3	1.022 Mg/m^3	0.986 Mg/m^3
Absorption coefficient	1.153 mm^{-1}	3.745 mm^{-1}	3.640 mm^{-1}
F(000)	1708	1614	1588
Crystal size (mm^3)	0.200 x 0.200 x 0.100	0.200 x 0.050 x 0.010	0.100 x 0.050 x 0.010
Theta range for data collection	3.117 to 74.931°	2.621 to 74.814°	2.247 to 76.128°
Index ranges	-21<=h<=21,<br>-24<=k<=24,<br>-16<=l<=18	-19<=h<=19,<br>-21<=k<=20,<br>-24<=l<=25	-19<=h<=18,<br>-20<=k<=21,<br>-24<=l<=25
Reflections collected	171919	160600	178437
Independent reflections	16964 [R(int) = 0.0884]	17110 [R(int) = 0.0655]	16106 [R(int) = 0.0884]
Completeness to theta = 67.684°	99.7 %	99.8 %	99.7 %
Refinement method	Full-matrix least-squares on F^2	Full-matrix least-squares on F^2	Full-matrix least-squares on F^2
Data / restraints / parameters	19192 / 1 / 1034	20304 / 29 / 854	20535 / 4 / 705
Goodness-of-fit on F2	1.036	1.042	1.620
Final R indices [I>2sigma(I)]	R_1 = 0.0979,<br>wR_2 = 0.2680	R_1 = 0.0783,<br>wR_2 = 0.2138	R_1 = 0.1234,<br>wR_2 = 0.3533
R indices (all data)	R_1 = 0.1031,<br>wR_2 = 0.2735	R_1 = 0.0868,<br>wR_2 = 0.2232	R_1 = 0.1402,<br>wR_2 = 0.3764
Extinction coefficient	n/a	n/a	n/a
Largest diff. peak and hole	3.688 and -1.097 e·Å^{-3}	3.745 and -1.121 e·Å^{-3}	5.955 and -2.116 e·Å^{-3}

JUK-20(Zn) (CCDC 2225152):

Data collection and reduction

Diffraction data for a single crystal were collected at 100 K using the Rigaku Oxford Diffraction Synergy-S four circle diffractometer, equipped with the Cu (1.54184 Å) Kα radiation source, graphite monochromator, and CryoStream system for measurements at low temperature. Cell refinement and data reduction was performed using the CrysAlis Pro firmware.[8]

Structure solution and refinement

The phase problem was solved by direct methods and positions of all non-hydrogen atoms were determined using SHELXT program.[9] All non-hydrogen atoms were refined anisotropically using weighted full-matrix least-squares on F^2. Refinement and additional calculations were carried out using SHELXL-2018.[10] All programs used are components of the WINGX.[11]

Hydrogen atoms treatment

All hydrogen atoms joined to carbon atoms were positioned with an idealized geometry and refined using a riding model with Uiso(H) fixed at 1.5 Ueq of methyl C and 1.2 of other C atoms.

Hydrogen atoms joined to: N2, N3, N17 and N18 were also positioned with an idealized geometry like in aromatics and refined using a riding model with Uiso(H) fixed at 1.2 Ueq of the origin nitrogen atom (AFIX 43).

Hydrogen atoms of water molecule, joined to O16 atom, were found based on the differential Fourier map.

Notes

Part of the solvent DMF and water molecules, which fill the channels along the [001] direction, are unreachable from the Fourier difference map, with exceptions for six of them that are included in the structure model. The PLATON SQUEEZE[12] procedure was used to avoid problems with disordered DMF molecules and to improve the structure refinement process.

JUK-20(Cd)-ala (CCDC 2225153):

Data collection and reduction

Diffraction data for a single crystal were collected at 100 K using the Rigaku Oxford Diffraction Synergy-S four circle diffractometer, equipped with the Cu (1.54184 Å) Kα radiation source, graphite monochromator, and CryoStream system for measurements at low temperature. Cell refinement and data reduction was performed using the CrysAlis Pro firmware.[8]

Structure solution and refinement

The phase problem was solved by direct methods and positions of all non-hydrogen atoms were determined using SHELXT program.[9] Majority of non-hydrogen atoms were refined anisotropically using weighted full-matrix least-squares on F^2, with the only exception for dpt-ala ligand core (refined isotropically). Refinement and further calculations were carried out using SHELXL-2018.[10] All programs used are components of the WINGX.[11] A very strong positional/rotational disorder of the dpt-ala ligand resulted in the difficulties in refinement process, therefore, an attempt to disorder modelling was made. The central diazine aromatic rings (C44-C49 and N88-C92 atoms) were divided into two parts of inequal occupancies. Only for the more abundant ring was the substituent hydroxymethyl group found and refined (C50, O51). Due to the strong structural disorder, DFIX, DANG and EADP constraints were applied to ensure the convergence of the process of refining the structure model and the correct geometry of the molecule.

Hydrogen atoms treatment

All hydrogen atoms joined to carbon atoms were positioned with an idealized geometry and refined using a riding model with Uiso(H) fixed at 1.5 Ueq of methyl C and 1.2 of other C atoms.

Hydrogen atoms joined to: N11, N13, N37 and N39 were also positioned with an idealized geometry as in aromatics and refined using a riding model with Uiso(H) fixed at 1.2 Ueq of origin nitrogen atom (AFIX 43).

Hydrogen atoms of water molecule, joined to O45 atom, were found based on differential Fourier map.

Notes

Part of the solvent DMF and water molecules, which fill the channels along the [001] direction, are unreachable from the Fourier difference map, with exceptions for six of them that are included in the structure model. The PLATON SQUEEZE[12] procedure was used to avoid problems with disordered DMF molecules and to improve the structure refinement process.

JUK-20(Cd)-nol (CCDC 2225154):

Data collection and reduction

Diffraction data for a single crystal were collected at 100 K using the Rigaku Oxford Diffraction Synergy-S four circle diffractometer, equipped with the Cu (1.54184 Å) Kα radiation source, graphite monochromator, and CryoStream system for measurements at low temperature. Cell refinement and data reduction was performed using the CrysAlis Pro firmware.[8]

Structure solution and refinement

The phase problem was solved by direct methods and positions of all non-hydrogen atoms were determined using SHELXT program.[9] Majority of non-hydrogen atoms were refined anisotropically using weighted full-matrix least-squares on F^2, with the only exception for dpt-nol ligand core (refined isotropically). Refinement and further calculations were carried out using SHELXL-2018.[10] All programs used are components of the WINGX.[11] A very strong positional/rotational disorder of the dpt-nol ligand resulted in the difficulties in refinement process; therefore, an attempt to disorder modelling was made. Due to the strong structural disorder, DFIX, DANG and EADP constraints were applied to ensure the convergence of the process of refining the structure model and the correct geometry of the molecule.

Hydrogen atoms treatment

All hydrogen atoms joined to carbon atoms were positioned with an idealized geometry and refined using a riding model with Uiso(H) fixed at 1.5 Ueq of methyl C and 1.2 of other C atoms.

Hydrogen atoms joined to: N6, N7, N10 and N13 were also positioned with an idealized geometry like in aromatics and refined using a riding model with Uiso(H) fixed at 1.2 Ueq of the origin nitrogen atom (AFIX 43).

Notes

Part of the solvent DMF and water molecules, which fill the channels along the [001] direction, are unreachable from the Fourier difference map, with exceptions for six of them that are included in the structure model. The PLATON SQUEEZE[12] procedure was used to avoid problems with disordered DMF molecules and to improve the structure refinement process.

Presumably, because of the high value of the absorption coefficient and the highly disordered nature of water molecules, the position of hydrogen atoms next to oxygen atoms, which are water molecules in the studied structures, could not be determined from the differential Fourier map, so category B alerts were generated during data validation (checkcif).

Alert type "PLAT973_ALERT_2_A Check Calcd Positive Resid" for all structures is explained in the literature and in crystallographic forums by several phenomena, e.g. non-merohedral twinning (for all described structures there are no other warning signals indicating twinning), high X-ray absorption due to the

presence of a heavy atom in the structure. Attempts to model the disorder of Zn or Cd atoms did not yield positive results, and subsequent iterations made the structure model unstable. Another reason for the appearance on the differential Fourier map of relatively large residual peaks near the heavy atom may be the so-called Fourier series cut-off, which manifests itself in a symmetric and close distribution of peaks around the heavy atom. The appearance of the aforementioned peaks also predicts what has long been observed and described (for details, see the monograph "Inorganic Molecular Dissymmetry," Saito Y. 1979, Springer-Verlag Berlin Heidelberg New York) the ligand field theory for d electrons in non-bonding orbitals. Another reason for the appearance of residual peaks in the vicinity of a heavy atom, which is considered in the literature, is an erroneous spherical description of the electron density distribution around such an atom. In our opinion, due to the fact that a new formalism for aspherical scattering coefficients has been implemented in the SHELXL least squares crystallographic refinement program since 2018, and due to the fact that structure 6 was refined based on SHELXL-2018, the latter reason does not apply. The introduced formalism is based on Gaussian functions and can optionally supplement the independent atom model to account for the deformation of the electron density distribution due to chemical bonding and lone pairs. The asphericity contributions were derived from the electron density obtained from quantum chemical density functional theory calculations of the corresponding model compounds. As a result, the structure refinement results obtained by the least squares method are significantly improved after applying the new model.

In our opinion, the reason for the appearance of residual peaks at a distance of about 1 Å from the heavy atom is the large value of the absorption coefficient and the Fourier series cut-off. In the case of the studied structures, these reasons seem to be the most reasonable and are the cause of the A alert in the checkcif for all three structures. It should also be borne in mind that it is not always possible to obtain satisfactory measurements, which is due, for example, to the fact that on the basis of X-ray diffraction measurements on a monocrystal we obtain time-averaged data with all the defects and imperfections of the crystal, which ultimately affect the final result.

References

(1) Jędrzejowski, D.; Pander, M.; Nitek, W.; Bury, W.; Matoga, D. Turning Flexibility into Rigidity: Stepwise Locking of Interpenetrating Networks in a MOF Crystal through Click Reaction. *Chem. Mater.* **2021**, *33* (18), 7509–7517. https://doi.org/10.1021/acs.chemmater.1c02451.

(2) Brown, R. F. C.; Coulston, K. J.; Eastwood, F. W.; Gatehouse, B. M.; Guddatt, L. W.; Pfenninger, M.; Rainbow, I. Synthesis of Precursors of C4 and C5 Cumulenones. *Aust. J. Chem.* **1984**, *37* (12), 2509–2524. https://doi.org/10.1071/ch9842509.

(3) Sarotti, A. M.; Pisano, P. L.; Pellegrinet, S. C. A Facile Microwave-Assisted Diels–Alder Reaction of Vinylboronates. *Org. Biomol. Chem.* **2010**, *8* (22), 5069–5073. https://doi.org/10.1039/C0OB00020E.

(4) Rouquerol, J.; Llewellyn, P.; Rouquerol, F. Is the BET Equation Applicable to Microporous Adsorbents? In *Studies in surface science and catalysis*; Elsevier B. V., 2007; Vol. 160, pp 49–56. https://doi.org/10.1016/S0167-2991(07)80008-5.

(5) Walton, K. S.; Snurr, R. Q. Applicability of the BET Method for Determining Surface Areas of Microporous Metal-Organic Frameworks. *J. Am. Chem. Soc.* **2007**, *129* (27), 8552–8556. https://doi.org/10.1021/ja071174k.

(6) Willems, T. F.; Rycroft, C. H.; Kazi, M.; Meza, J. C.; Haranczyk, M. Algorithms and Tools for High-Throughput Geometry-Based Analysis of Crystalline Porous Materials. *Microporous Mesoporous Mater.* **2012**, *149* (1), 134–141. https://doi.org/10.1016/j.micromeso.2011.08.020.

(7) Ongari, D.; Boyd, P. G.; Barthel, S.; Witman, M.; Haranczyk, M.; Smit, B. Accurate Characterization of the Pore Volume in Microporous Crystalline Materials. *Langmuir* **2017**, *33* (51), 14529–14538. https://doi.org/10.1021/acs.langmuir.7b01682.

(8) Rigaku Oxford Diffraction, 2018, CrysAlisPro Software System, Version 1.171.40.67a, Rigaku Corporation, Oxford, UK.

(9) Sheldrick, G. M. SHELXT – Integrated Space-Group and Crystal-Structure Determination. *Acta Crystallogr. Sect. Found. Adv.* **2015**, *71* (1), 3–8. https://doi.org/10.1107/S2053273314026370.

(10) Sheldrick, G. M. Crystal Structure Refinement with SHELXL. *Acta Crystallogr. Sect. C Struct. Chem.* **2015**, *71* (1), 3–8. https://doi.org/10.1107/S2053229614024218.

(11) Farrugia, L. J. WinGX and ORTEP for Windows: An Update. *J. Appl. Crystallogr.* **2012**, *45* (4), 849–854. https://doi.org/10.1107/S0021889812029111.

(12) Spek, A. L. PLATON SQUEEZE: A Tool for the Calculation of the Disordered Solvent Contribution to the Calculated Structure Factors. *Acta Crystallogr. Sect. C Struct. Chem.* **2015**, *71* (Pt 1), 9–18. https://doi.org/10.1107/S2053229614024929.
